# Study of Pentacyclic Triterpenes from Lyophilised Aguaje: Anti-Inflammatory and Antioxidant Properties

**DOI:** 10.3390/ijms25179615

**Published:** 2024-09-05

**Authors:** Luis Apaza Ticona, Javier Sánchez Sánchez-Corral, Natalia Montoto Lozano, Pablo Prieto Ramos, Ángel Rumbero Sánchez

**Affiliations:** 1 Organic Chemistry Unit, Department of Chemistry in Pharmaceutical Sciences, Faculty of Pharmacy, University Complutense of Madrid, Plza. Ramón y Cajal s/n, 28040 Madrid, Spain; 2 Department of Organic Chemistry, Faculty of Sciences, University Autónoma of Madrid, Cantoblanco, 28049 Madrid, Spain

**Keywords:** Mauritia, triterpenes, inflammation, antioxidant, NF-κB, NO, SOD, Nrf2

## Abstract

*Mauritia flexuosa (M. flexuosa)*, commonly known as Aguaje or Moriche palm, is traditionally recognised in South America for its medicinal properties, particularly for its anti-inflammatory and antioxidant effects. However, the bioactive compounds responsible for these effects have not been thoroughly investigated. This study aims to isolate and characterise pentacyclic triterpenoid compounds from *M. flexuosa* and to evaluate their therapeutic potential. Using various chromatographic and spectroscopic techniques including Nuclear Magnetic Resonance (NMR) and Mass Spectrometry (MS), three pentacyclic triterpenoid compounds were successfully isolated. Among them, compound **1** (3,11-dioxours-12-en-28-oic acid) exhibited notable bioactivity, significantly inhibiting the activation of Nuclear Factor kappa-light-chain-enhancer of activated B cells (NF-κB) (IC_50_ = 7.39–8.11 μM) and of Nitric Oxide (NO) (IC_50_ = 4.75–6.59 μM), both of which are key processes in inflammation. Additionally, compound **1** demonstrated potent antioxidant properties by activating the antioxidant enzyme Superoxide Dismutase (SOD) (EC_50_ = 1.87 μM) and the transcription factor Nuclear factor erythroid 2-related factor 2 (Nrf2) (EC_50_ = 243–547.59 nM), thus showing its potential in combating oxidative stress. This study is the first to isolate and characterise the three compounds from *M. flexuosa*, suggesting that compound **1** could be a promising candidate for the development of safer and more effective therapies for inflammatory and oxidative stress-related diseases.

## 1. Introduction

Inflammation is a complex physiological response of the body to harmful stimuli, such as infections, injuries, or stress [1]. When this response becomes chronic or dysregulated, it can have detrimental consequences for health, affecting various pharmacological targets such as the nuclear factor kappa-light-chain-enhancer of activated B cells (NF-κB), nitric oxide (NO), nuclear factor erythroid 2-related factor 2 (Nrf2), and superoxide dismutase (SOD).

In this regard, the prolonged activation of NF-κB, triggered by stimuli such as proinflammatory cytokines and reactive oxygen species (ROS), leads to the overexpression of proinflammatory genes, exacerbating inflammation and contributing to tissue damage [2]. Once activated, NF-κB translocates to the cell nucleus and initiates the expression of proinflammatory genes such as tumour necrosis factor-α (TNF-*α*), interleukin-1*β* (IL-1*β*), and interleukin-6 (IL-6). Additionally, other proteins such as IκB kinases (IKKs) phosphorylate IκB, releasing NF-κB for its activation and translocation to the nucleus [3].

Moreover, during inflammation, the expression of the enzyme nitric oxide synthase (NOS), especially the inducible nitric oxide synthase isoform (iNOS), increases, leading to an overproduction of NO. The excess NO can react with ROS to form peroxynitrite (ONOO^−^), a highly reactive free radical that increases oxidative stress and inflammation. Additionally, NO can induce mitochondrial dysfunction and cellular apoptosis, thereby contributing to tissue damage [4].

Furthermore, during inflammation, the Nrf2 signalling pathway may be suppressed due to the activation of proinflammatory transcription factors such as NF-κB, which inhibit the expression of antioxidant genes. Additionally, ROS generated during the inflammatory response can oxidise cysteine residues in the Nrf2-Kelch-like ECH-associated protein 1 (Keap1) regulator, releasing Nrf2 for translocation to the cell nucleus. However, Nrf2 activity may be counteracted by the activity of a variety of kinases, including IKK, which phosphorylate Nrf2 and direct it towards proteasomal degradation, thereby limiting its ability to counteract oxidative stress and inflammation [5].

Finally, during chronic inflammation, SOD activity may be compromised due to depletion of antioxidant substrates such as glutathione or inhibition by ROS generated during the inflammatory response. Additionally, SOD expression may be regulated by NF-κB and Nrf2. On one hand, NF-κB overexpression may inhibit SOD expression, exacerbating oxidative stress and inflammation. On the other hand, Nrf2 can induce SOD expression and other antioxidant enzymes, thereby enhancing the cell’s antioxidant capacity and reducing oxidative damage [6].

Although there are a series of treatments targeting the NF-κB, NO, SOD, and Nrf2 pharmacological targets, their clinical outcomes have proved a series of limitations. Inhibitors of NF-κB activation show serious side effects and different levels of efficacy in chronic inflammatory diseases [7]. In the case of NO inhibitors, they may have undesired systemic effects on cardiovascular function, neurotransmission, and other physiological processes [8]. Moreover, agents activating Nrf2, such as bardoxolone, have shown questionable clinical efficacy, as evidenced by studies showing an increased risk of cardiovascular events [9,10]. Finally, SOD mimetics that enhance SOD activity have exhibited limited clinical efficacy, as seen in clinical trials that have not shown significant benefits in neurodegenerative diseases, such as Alzheimer’s disease [11].

Natural products derived from plants have long been an invaluable source of bioactive compounds with anti-inflammatory and antioxidant properties [12]. These compounds have shown beneficial effects on human health by modulating various cellular signalling pathways and counteracting oxidative stress. Numerous studies have demonstrated that various natural products, such as curcumin (10–50 μM) and quercetin (25–100 μM), are capable of inhibiting NF-κB activation [13,14]. Additionally, extracts of green tea (25–100 μg mL^−1^) and grape seed (10–50 μg mL^−1^) have shown the ability to inhibit NO production [15,16]. Moreover, it has been found that certain natural products, such as sulforaphane (5–20 μM), which is present in broccoli, can activate the Nrf2 signalling pathway [17]. Finally, it has been demonstrated that natural products such as the polyphenols in green tea (10–50 μg mL^−1^) significantly increase SOD activity [18].

The *Mauritia flexuosa* L. f. (*M. flexuosa*) species is part of the Arecaceae family. It is a palm tree endemic to South America, known by various names such as Aguaje (Peru), Buriti (Brazil), Moriche Palm (Venezuela, Bolivia, Colombia, Panama, and Puerto Rico), and Morete (Ecuador) [19,20]. Since ancient times, communities in Colombia like the Huitoto have used the roots of this plant in baths for arthritis patients or those with body pains [21]. Moreover, a recent study has proven the anti-inflammatory activity (inhibition of IL-1*β* and TNF-α) of the epicarp, mesocarp, and endocarp of the *M. flexuosa* fruit at a dose of 1000 mg kg^−1^ in a murine model [22].

Regarding the phytochemical composition of *M. flexuosa*, previous studies have isolated and analysed phytosterols such as stigmasterol, *β*-sitosterol, campesterol, and stigmastan-3,5-diene from the oil of *M. flexuosa* [23,24]. Additionally, triterpenes have been analysed from the *n*-hexane extract of *M. flexuosa* roots, including friedeline, taraxerone, and lupenyl acetate, as well as lupenone, betulin, and betulinic acid [25,26].

The current article shows how pentacyclic triterpenes have been isolated, while also analysing their composition. This is followed by an analysis of the anti-inflammatory (inhibition of NF-κB and NO) and antioxidant (activation of SOD and Nrf2) activities of the isolated compounds.

## 2. Results

**Figure 1:**
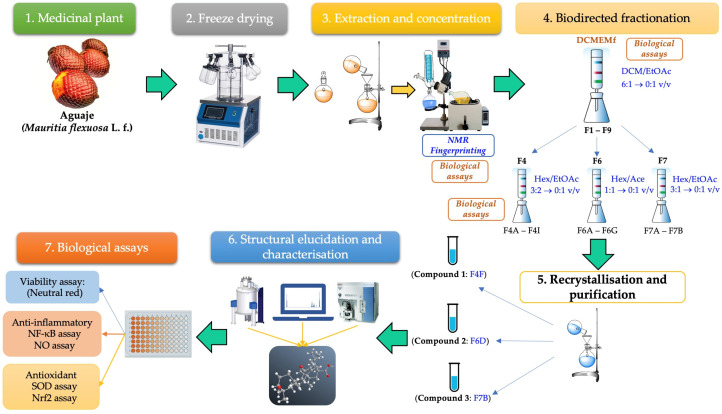
Comprehensive research process: plant treatment, extraction, purification, and biological assays of compounds.

To understand the results of our study, Figure 1 provides a schematic overview of the steps undertaken to isolate compounds with anti-inflammatory and antioxidant activity. The diagram covers the process from plant treatment to extraction, purification, and compound characterisation.

### 2.1. Isolation and Characterisation of Compounds of M. flexuosa

After two extractions from the lyophilised extract of *M. flexuosa* (Figure S1), an analysis of the ^1^H NMR and ^13^C NMR spectra was carried out. In the ^1^H NMR spectra of the *n*-hexane extract of *M. flexuosa* (HEMf), a significant number of aliphatic signals (between 0.8 and 2.2 ppm) were observed, which likely corresponded to essential oils. Most of these are hydrocarbon compounds with limited functional groups (Figure S2). Furthermore, the ^13^C NMR spectrum of HEMf showed characteristic signals of *sp^3^* and *sp^2^* aromatic carbons with no apparent binding to heteroatoms (Figure S3).

The ^1^H NMR spectra of the dichloromethane/methanol extract of *M. flexuosa* (DCMEMf) displayed a higher number of allylic (5.0–5.7 ppm) and aromatic (6.8–7.1 ppm) signals, suggesting the presence of more complex chemical structures with potential biological activity (Figure S4). Based on these results, the DCMEMf was fractionated, leading to the isolation and characterisation of three compounds (Figure 2 and Figures S6–S14).

Almost all the signals of the HEMf corresponded to nonpolar compounds, such as sesquiterpenoids or essential oils, which could be observed in the initial ranges of the spectrum (Figures S2 and S3). Notably, *M. flexuosa* is primarily composed of phytosterols and tocopherols [23]. In contrast, the DCMEMf exhibited characteristic signals of triterpenes with an ursane-type skeleton (Figures S4 and S5).

### 2.2. Viability Assay of the Extracts and Compounds of M. flexuosa

**Figure 3:**
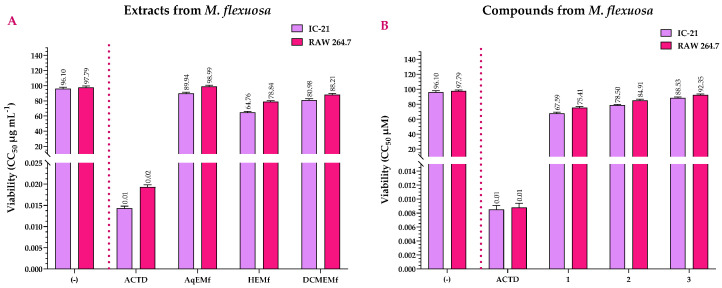
CC_50_ values of the NR (Viability) assays calculated for the extracts (**A**) and compounds (**B**) of *M. flexuosa* at 72 h. (−) = untreated cells (negative control); ACTD = actinomycin D (positive control); AqEMf = aqueous extract of *M. flexuosa*; HEMf = *n*-hexane extract of *M. flexuosa*; DCMEMf = dichloromethane/methanol extract of *M. flexuosa*. Viability CC_50_ values represent the mean of three independent assays.

Figure 3A shows the cell viability results of *M. flexuosa* extracts, along with actinomycin D (ACTD, positive control) and untreated cells (negative control). ACTD exhibited CC_50_ values of 0.0143 and 0.0193 μg mL^−1^ for the IC-21 and RAW 264.7 cell lines, respectively. These values were considered cytotoxic as they showed statistically significant differences (*p* < 0.001) compared to the negative control (Table S1). Regarding the extracts, only AqEMf did not show statistically significant differences with the negative control in the RAW 264.7 (*p* = 0.43) cell line, showing that this extract was not cytotoxic in this cell line (Table S1). Furthermore, all extracts had statistically significant differences (*p* < 0.001) with ACTD. This shows that AqEMf and DCMEMf were slightly cytotoxic when compared to ACTD in IC-21 and RAW 264.7 cell lines, with CC_50_ values of 89.94 and 98.99 μg mL^−1^ for AqEMf, and 80.98 and 88.21 μg mL^−1^ for DCMEMf, respectively (Table S1). In contrast, although HEMf showed slight cytotoxicity when compared to ACTD, it exhibited moderate cytotoxicity when compared to AqEMf and DCMEMf in IC-21 and RAW 264.7 cell lines, with CC_50_ values of 64.76 and 78.84 μg mL^−1^, respectively (Figure 3A and Table S1).

In relation to the compounds, all of them showed statistically significant differences (*p* < 0.001) when compared to the positive (ACTD, CC_50_ = 0.0085–0.0088 μM) and negative (untreated cells) controls (Table S2). These results show that although all three compounds were slightly cytotoxic, their cytotoxicity was not higher than that of ACTD, with CC_50_ values of 67.59, 78.50, and 88.53 μM for the IC-21 cell line; and 75.41, 84.91, and 92.35 μM for RAW 264.7, respectively. However, compound **1** had moderate cytotoxicity when compared to compounds **2** and **3** in all tested cell lines (Figure 3B and Table S2).

### 2.3. Anti-Inflammatory Activity of the Extracts and Compounds of M. flexuosa

**Figure 4:**
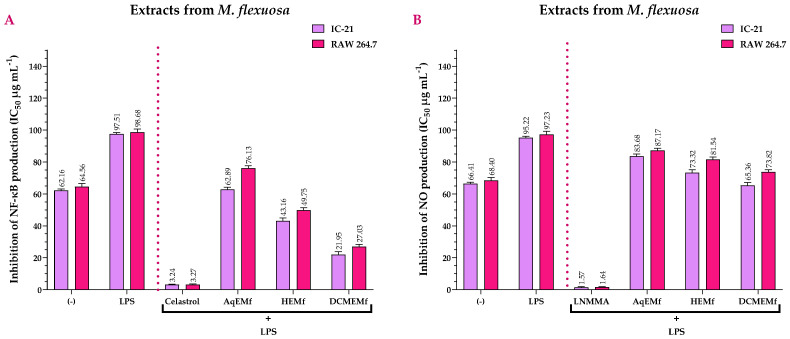
Effects of *M. flexuosa* extracts on NF-κB (**A**) and NO (**B**) production induced by LPS at 72 h. (−) = untreated cells (negative control); LPS = Group of cells treated with 100 μg mL^−1^ of LPS; CEL = celastrol (positive control); LNMMA = N^G^-methyl-L-arginine acetate salt (positive control); AqEMf = aqueous extract of *M. flexuosa*; HEMf = *n*-hexane extract of *M. flexuosa*; DCMEMf = dichloromethane/methanol extract of *M. flexuosa*. NF-κB (Nuclear Factor kappa-light-chain-enhancer of activated B cells) and NO (Nitric Oxide) IC_50_ values represent the mean of three independent assays.

Figure 4A compares the IC_50_ values of the NF-κB inhibition assay (stimulated with lipopolysaccharide, LPS) of *M. flexuosa* extracts with celastrol (CEL, positive control). CEL had IC_50_ values of 3.24 and 3.27 µg mL^−1^ in the IC-21 and RAW 264.7 cell lines, respectively. All extracts showed statistically significant differences (*p* < 0.001) compared to the CEL. This shows that while the extracts had anti-NF-κB potential, their activity was not higher than that of CEL (Table S3). However, when comparing the IC_50_ values of NF-κB inhibition among the extracts, significant statistical differences were observed (*p* < 0.001). DCMEMf had higher anti-inflammatory (NF-κB inhibition) activity than AqEMf and HEMf, with IC_50_ values of 21.95 and 27.03 µg mL^−1^ in the IC-21 and RAW 264.7 cell lines, respectively (Table S3).

Similarly, Figure 4B depicts the NO inhibition (stimulated with LPS) of the *M. flexuosa* extracts in comparison to N^G^-methyl-L-arginine acetate salt (LNMMA, positive control). LNMMA recorded IC_50_ values of 1.57 µg mL^−1^ for IC-21 cells and 1.64 µg mL^−1^ for RAW 264.7 cells. All extracts showed statistically significant differences (*p* < 0.001) when compared to LNMMA. This shows that, although the extracts exhibited anti-NO potential, their activity did not exceed that of LNMMA (Table S4). However, comparing the IC_50_ values for NO inhibition across the extracts revealed significant statistical differences (*p* < 0.001). DCMEMf exhibited higher anti-inflammatory (NO inhibition) activity than AqEMf and HEMf, with IC_50_ values of 65.36 and 73.82 µg mL^−1^ in the IC-21 and RAW 264.7 cell lines, respectively (Table S4).

**Figure 5:**
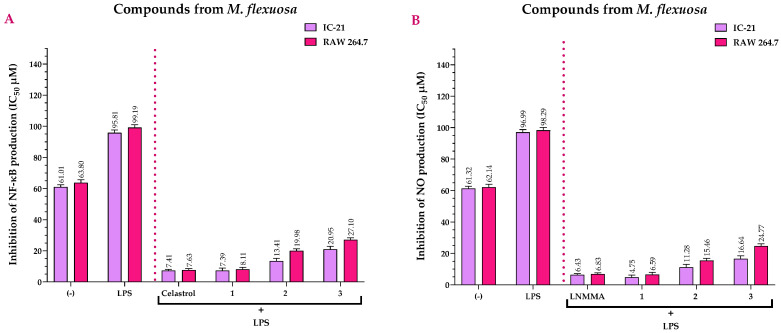
Effects of *M. flexuosa* compounds on NF-κB (**A**) and NO (**B**) production induced by LPS at 72 h. (−) = untreated cells (negative control); LPS = Group of cells treated with 100 μg mL^−1^ of LPS; CEL = celastrol (positive control); LNMMA = N^G^-methyl-L-arginine acetate salt (positive control). NF-κB (Nuclear Factor kappa-light-chain-enhancer of activated B cells) and NO (Nitric Oxide) IC_50_ values represent the mean of three independent assays.

Figure 5A compares the IC_50_ values for NF-κB inhibition (stimulated with LPS) of the compounds with those of CEL, which showed IC_50_ values of 7.41 µM in IC-21 cell lines and 7.63 µM in RAW 264.7 cell lines. Compound **1** did not show statistically significant differences when compared to CEL in the IC-21 (*p* > 0.99, IC_50_ = 7.39 µM) and RAW 264.7 (*p* = 0.97, IC_50_ = 8.11 µM) cell lines (Table S5), thus showing that they have similar anti-inflammatory potential. In contrast, compounds **2** and **3** showed statistically significant differences (*p* < 0.001) when compared to CEL in both the IC-21 and RAW 264.7 cell lines, thus exhibiting moderate anti-inflammatory activity (Figure 5A and Table S5).

In contrast, Figure 5B shows the NO inhibition (stimulated with LPS) of the *M. flexuosa* compounds compared to LNMMA. LNMMA recorded IC_50_ values of 6.43 µM in IC-21 cells and 6.83 µM in RAW 264.7 cells. Compound **1** showed no statistically significant differences with LNMMA in the IC-21 (*p* = 0.21, IC_50_ = 4.75 µM) and RAW 264.7 (*p* > 0.99, IC_50_ = 6.59 µM) cell lines (Table S6), suggesting a similar anti-inflammatory effect. On the other hand, compounds **2** and **3** had statistically significant differences (*p* < 0.001) with LNMMA in both the IC-21 and RAW 264.7 cell lines, showing moderate anti-inflammatory activity (Figure 5B and Table S6).

### 2.4. Antioxidant Activity of the Extracts and Compounds of M. flexuosa

**Figure 6:**
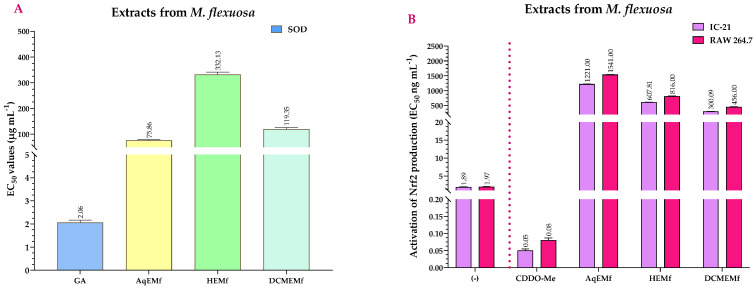
Effects of *M. flexuosa* extracts on SOD (**A**) and Nrf2 (**B**) activation at 72 h. (−) = untreated cells (negative control); GA = gallic acid (positive control); CDDO-Me = bardoxolone methyl (positive control); AqEMf = aqueous extract of *M. flexuosa*; HEMf = *n*-hexane extract of *M. flexuosa*; DCMEMf = dichloromethane/methanol extract of *M. flexuosa*. SOD (Superoxide Dismutase) and Nrf2 (Nuclear factor erythroid 2-related factor 2) EC_50_ values represent the mean of three independent assays.

EC_50_ values for the SOD activity assay of *M. flexuosa* extracts, as depicted in Figure 6A, were compared to those of gallic acid (GA), which served as the positive control with an EC_50_ of 2.06 µg mL^−1^. The data revealed that, although the extracts had SOD activity, their EC_50_ values were significantly higher than that of GA, therefore they were less effective. Additionally, when comparing the extracts, AqEMf and DCMEMf showed superior SOD activity when compared to HEMf (*p* < 0.001), with EC_50_ values of 75.86 µg mL^−1^ and 119.35 µg mL^−1^, respectively (Table S7).

Figure 6B shows the EC_50_ values for the Nrf2 activation assay for the extracts from *M. flexuosa* compared to bardoxolone methyl (CDDO-Me, positive control), which had an EC_50_ value of 0.05 and 0.08 ng mL^−1^ in the IC-21 and RAW 264.7 cell lines, respectively. All extracts showed statistically significant differences (*p* < 0.001) when compared to CDDO-Me, showing that although they activated Nrf2, their EC_50_ values were higher, making them less effective. Furthermore, among the extracts, HEMf and DCMEMf showed higher Nrf2 activation activity than AqEMf (*p* < 0.001), with EC_50_ values of 607.81 ng mL^−1^ in the IC-21 cells and 816.00 ng mL^−1^ in the RAW 264.7 cells for HEMf, and 300.09 ng mL^−1^ in the IC-21 cells and 456.00 ng mL^−1^ in the RAW 264.7 cells for DCMEMf (Table S8).

**Figure 7:**
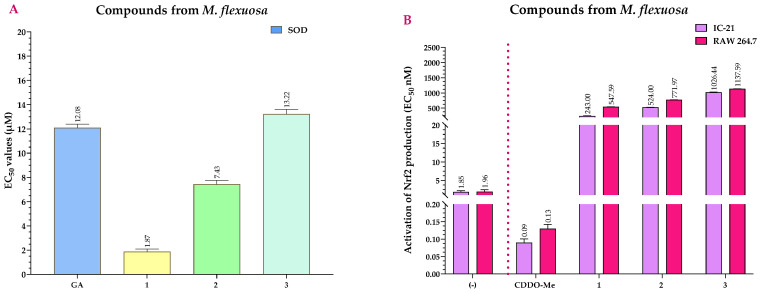
Effects of *M. flexuosa* compounds on SOD (**A**) and Nrf2 (**B**) activation at 72 h. (−) = untreated cells (negative control); GA = gallic acid (positive control); CDDO-Me = bardoxolone methyl (positive control). SOD (Superoxide Dismutase) and Nrf2 (Nuclear factor erythroid 2-related factor 2) EC_50_ values represent the mean of three independent assays.

Similarly, Figure 7A compares the EC_50_ values for SOD activity of the compounds with GA. The EC_50_ value of GA was 12.08 μM. All compounds showed statistically significant differences (*p* > 0.001) when compared to GA, showing relevant SOD activity. However, compounds **1** (EC_50_ = 1.87 μM) and **2** (EC_50_ = 7.43 μM) (*p* > 0.001), with lower EC_50_ values than GA, were more effective than compound **3** (EC_50_ = 13.22 μM) which had a slightly higher EC_50_ than GA (Table S9).

Regarding Nrf2 activation, although compounds **1**, **2**, and **3** had statistically significant differences (*p* > 0.001) when compared to CDDO-Me (EC_50_ = 0.09–0.13 nM), their EC_50_ values were higher, therefore having less Nrf2 activation potential than CDDO-Me (Figure 7B). However, when comparing the agonist activity among the compounds, compound **1** was the most active, with EC_50_ values of 243 nM (IC-21 cells) and 547.59 nM (RAW 264.7 cells) (Table S10).

## 3. Discussion

This section examines our findings on compound identification, the cytotoxicity of extracts and compounds, and their anti-inflammatory and antioxidant implications, specifically through NF-κB inhibition and NO production, as well as SOD activation and Nrf2. We compare our results with previous studies to provide a broader context and validate our observations.

Firstly, we identified pentacyclic triterpenes in the aqueous extract of *M. flexuosa* (AqEMf) for the first time, offering new insights into the chemical composition of this plant. These compounds had previously been identified in other plant species, such as compound **1** in *Ilex rotunda* [27], compound **2** in *Diospyros decandra* [28], and compound **3** in *Cyclocarya paliurus* [29]. Describing these compounds in *M. flexuosa* significantly expands the known pharmacological potential of this plant species, suggesting its potential for developing new therapeutic agents, particularly due to the diverse biological activities associated with pentacyclic triterpenes, such as anti-inflammatory, anti-cancer, and anti-viral properties [30].

After identifying the compounds present in the *M. flexuosa* extracts, we assessed the safety profile of the extracts and isolated compounds through cytotoxicity determination. Cytotoxicity assays showed that AqEMf, DCMEMf, and HEMf exhibited different degrees of cytotoxicity, although they had less cytotoxicity than ACTD (positive control). Comparing the three extracts, HEMf had higher cytotoxicity than the other two extracts in both IC-21 and RAW 264.7 cell lines. This increase in cytotoxicity can be attributed to the *n*-hexane treatment of the roots of *M. flexuosa*, which extracts lipidic compounds with higher lipophilicity, increasing permeability and thus cytotoxicity [31]. This finding underscores the importance of solvent selection in the extraction process, as it can significantly influence the cytotoxic profile of plant-derived compounds.

Regarding the isolated compounds, the pentacyclic triterpenoids obtained from DCMEMf showed lower cytotoxicity compared to ACTD. However, compound **1** had a higher cytotoxicity than compounds **2** and **3**, due to its high polarity (cLogP = 6.62) [32]. The high lipophilicity of compound **1** in HEMf also contributed to its higher permeability and cytotoxicity [33,34]. Nevertheless, none of the three compounds showed significant cytotoxicity at concentrations below 70 µM in the macrophage cell lines, allowing further biological activity experiments.

Subsequently, we evaluated the anti-inflammatory activity of the extracts and isolated compounds from *M. flexuosa*. Previous studies, such as that of Rodrigues et al. [35], demonstrated that the fruit extract of *M. flexuosa* inhibited the expression of several inflammatory cytokines, including TNF-α, IL-1*β*, NO, and NF-κB. Their experiments showed that an in vivo injection of 1000 mg kg^−1^ of *M. flexuosa* extract resulted in approximately a 50% reduction in oedema volume, a reduction comparable to that achieved with indomethacin (a widely used COX inhibitor) at a dose of 10 mg kg^−1^. These findings are consistent with our study results, where the extracts of *M. flexuosa* showed a significant decrease in the expression of pro-inflammatory cytokines, with DCMEMf showing the highest effect.

Regarding the anti-inflammatory activity of the isolated compounds, it is worth noting that these compounds have a structural relationship with ursolic acid, known for its notable anti-inflammatory potential. The structural similarities between the isolated compounds and ursolic acid suggest a shared mechanism of action, likely involving modulation of key inflammatory pathways such as NF-κB [36]. This similarity opens avenues for the development of targeted therapies based on structural analogues of ursolic acid. For example, Lei et al. [37] found that ursolic acid significantly reduced the expression of pro-inflammatory cytokines such as IL-1*β*, TNF-α, and IL-6 in mice at doses ranging from 0 to 32 µM. Similar findings were reported by Li et al. [38], who linked these effects to the TLR4 pathway. Furthermore, the administration of an NF-κB activator neutralised the effects of ursolic acid, further connecting it to anti-inflammatory responses. Wang et al. [39] confirmed these properties in diseases related to chronic inflammation, such as diabetes. Ramírez-Rodríguez et al. [40] confirmed the anti-inflammatory effects of a daily dose of 150 mg of ursolic acid in human patients.

To understand how the pentacyclic triterpenoids interact with inflammatory signalling pathways, it is essential to grasp the role of the NF-κB and NO targets in inflammation processes. Chronic and immune-mediated diseases, such as skin disorders and arthritis, are characterised by persistent inflammation, which plays a fundamental role in their pathogenesis. Inflammation, a complex biological response to harmful stimuli, is regulated by various molecular pathways, with the NF-κB pathway being a central orchestrator regulating the expression of genes involved in immune and inflammatory responses [41]. Additionally, NO, a key signalling molecule, significantly contributes to the inflammatory process in these diseases. Dysregulation in NF-κB activation and NO production can exacerbate inflammation, leading to tissue damage and disease progression [42].

In this regard, the isolated compounds interfere with the NF-κB signalling pathway through various mechanisms, such as blocking the activation of kinases involved in the phosphorylation and degradation of IκB, preventing the release of NF-κB and its translocation to the nucleus to activate the transcription of pro-inflammatory genes. Alternatively, the compounds directly interact with NF-κB subunits, suppressing the phosphorylation of p65 (RelA) [43,44] or hindering NF-κB translocation to the nucleus [45]. They also modulate the expression of NF-κB pathway inhibitors, such as IκBα, helping to maintain NF-κB inactive in the cytoplasm [46].

Similarly, inhibiting NO production is another crucial aspect of the anti-inflammatory activity of these isolated compounds. Pentacyclic triterpenoids may exert their anti-inflammatory effect by inhibiting NO production through multiple mechanisms [47]. Besides reducing iNOS expression at the transcriptional level by inhibiting NF-κB, these compounds may directly modulate the catalytic activity of iNOS by affecting its function and reducing oxidative stress associated with NO production, thereby limiting the inflammatory impact of NO [48,49,50]. Finally, the higher activity of compound **1** in NO production inhibition is attributed to its ability to interfere with multiple points in the NO production pathway, related to its unique chemical structure, including specific interactions with the active sites of iNOS [51].

To better elucidate the mechanism of anti-inflammatory activity, we explored the relationship between NF-κB inhibition and NO production. The results of NF-κB and NO inhibition assays suggest that NF-κB inhibition, a key regulator of the inflammatory response, can influence NO production, an important inflammatory mediator. Inhibition of NF-κB by pentacyclic triterpenoids may reduce the expression of pro-inflammatory genes controlled by this pathway, leading to a general decrease in the inflammatory response, as demonstrated by Katsuyama et al. [52]. Furthermore, since NF-κB regulates the expression of iNOS, responsible for NO production during inflammation, its inhibition by triterpenoid compounds results in a simultaneous reduction in NO production, as evidenced by Jia et al. [53].

When analysing the structure-activity relationship (SAR) of the isolated triterpenoid compounds and their impact on inflammation and oxidative stress, we observed that the oxidised ursane-type triterpenoids (derived from ursolic acid) exhibit anti-inflammatory activity, likely due to the presence of an oxo group at C-11 conjugated with an *α,β*-unsaturated double bond at C-12/C-13 [54]. These structural features facilitate Michael-type reactions when exposed to nucleophiles, making them more effective [46]. The higher activity of compound **1** in NF-κB inhibition may be attributed to its specific chemical structure, especially the oxo group at C-3, which interacts with specific amino acid residues in the Iκκ*β* active site and leads to inhibitory effects on cytokine production, as demonstrated by Harun et al. [55] and Veerappan et al. [56].

SAR experiments conducted by Lauria et al. used molecular docking assays to show that the Iκκ*β* binding site includes three hydrophobic pockets with a lateral H-bonding pocket. Their findings showed that tri- and tetracyclic structures have significant inhibitory interactions with the binding site, suggesting that pentacyclic triterpenes might also exhibit similar activity. They also observed that oxygenated radicals, which act as H-bond acceptors, exhibit stronger interactions than non-oxygenated radicals, evidencing that oxo groups play a crucial role in inhibitory activity [57], as was the case for compound **1** in our research.

Once the anti-inflammatory effect of the extracts and compounds from *M. flexuosa* was determined, we explored their antioxidant properties, considering the close relationship between inflammation and oxidative stress through various factors, such as SOD and Nrf2 [58]. Previous studies have shown that both aqueous and alcoholic extracts of *M. flexuosa* can attenuate acute and chronic inflammation and exhibit significant antioxidant potential [59,60]. In our case, both AqEMf and DCMEMf showed similar levels of Nrf2 activation. However, in terms of SOD inhibition, AqEMf showed less potency than DCMEMf.

For the studied compounds, SOD enzyme activation involves complex molecular processes affecting its catalytic activity. SOD is crucial for reducing oxidative stress in cells by converting the superoxide radical into molecular oxygen and hydrogen peroxide. Understanding how triterpenoids activate SOD involves considering interactions with specific amino acid residues in their active site, such as histidines, cysteines, or tyrosines. These interactions can induce conformational changes in the enzyme, enhancing its catalytic activity and modulating its ability to reduce oxidative stress [61]. Presenting oxo and acetyl groups at C-3, triterpenoid compounds **1** and **2** interact more directly with specific amino acid residues in SOD compared to the hydroxyl group of compound **3**. These interactions may increase the affinity of compounds **1** and **2** for SOD or stabilise an active conformation of the enzyme, resulting in higher catalytic activity compared to compound **3** [62].

Regarding Nrf2 activation, all three isolated compounds are Nrf2 inducers, with compound **1** being the most active due to the oxo group at C-3 [63]. These compounds can activate the Nrf2 pathway through complex molecular processes affecting the interaction between Nrf2 and its inhibitory protein, Keap1. These compounds may influence the release of Nrf2 from the Nrf2-Keap1 complex in the cytoplasm and its subsequent translocation to the nucleus, where it regulates the expression of antioxidant and detoxification genes [64]. Previous studies have shown that pentacyclic triterpenoid compounds can interact directly with specific amino acid residues in Keap1, such as critical cysteines responsible for Keap1′s inhibitory function on Nrf2 [65]. These compounds can induce conformational changes in Keap1, altering its ability to bind and retain Nrf2, potentially through modifications of Nrf2 ubiquitination or Keap1 stability, leading to Nrf2 release and nuclear translocation [66]. The higher activity of compound **1** compared to compounds **2** and **3** may be related to the presence of the oxo group in its structure. Previous research suggests that this oxo group facilitates specific interactions with amino acid residues in Keap1, increasing compound **1**’s affinity for Keap1 [67]. Additionally, the oxo group induces conformational changes in Keap1 that favour Nrf2 release [68]. The oxo group also confers additional antioxidant properties to compound **1**, enhancing its ability to activate the Nrf2 pathway [69].

In conclusion, the activation of both SOD enzyme and Nrf2 transcription factor by our isolated compounds suggests a potential correlation between these processes in cellular antioxidant response. Both SOD and Nrf2 are key components of the body’s antioxidant defence, working together to maintain cellular redox balance and protect against oxidative damage [70]. By activating both SOD and Nrf2, pentacyclic triterpenoid compounds exert a synergistic effect on cellular protection against oxidative stress [71].

The three terpenoids are structurally diverse specifically at the C-3 position. Based on the results of the biological assays, we can conclude that the presence of a ketone moiety at the C-3 position enhances the potency of the pentacyclic triterpene scaffold compared to its hydroxylated and acetylated analogues. Additionally, substitutions at the C-11 and C-28 positions are also crucial for the activity of this scaffold.

In this study, we present a novel approach to isolating and analysing pentacyclic triterpenes from *M. flexuosa* with a specific focus on their anti-inflammatory and antioxidant properties. Our experimental techniques leverage advanced chromatographic and spectroscopic analyses to accurately characterise the bioactive compounds. This approach not only enhances the precision of compound identification but also provides a deeper understanding of their biological activities. The main advantage of our methodology lies in its ability to selectively isolate and quantify triterpenes, enabling a more detailed analysis of their pharmacological potential. Moreover, by evaluating both anti-inflammatory and antioxidant activities in parallel, we offer a comprehensive assessment of the therapeutic potential of this plant species.

However, despite these innovations, this study has limitations. The in vitro nature of the assays may not fully replicate the complexity of in vivo systems, and the high concentrations required to observe significant biological effects may limit the direct clinical translation. Additionally, while the study provides valuable insights into the pharmacological properties of *M. flexuosa*, further research is needed to elucidate the underlying molecular mechanisms and to assess the bioavailability and toxicity of the isolated compounds in more complex biological systems.

## 4. Materials and Methods

### 4.1. Chemicals and Reagents

High-purity organic solvents, sourced from Merck, were used in the extraction and fractionation processes, as well as for the isolation of the compounds. Silica gel (SiO_2_ 60 GF254 Sigma-Aldrich, CAS No. 112926-00-8, St. Louis, MO, USA) was used to carry out thin-layer chromatography (TLC). The chromatographic samples were evaluated through two separate methods: a chemical approach using phosphomolybdic acid solution (12Mo_12_O_3_ • H_3_PO_4_ ≥ 99.99% Sigma-Aldrich, CAS No. 51429-74-4, St. Louis, MO, USA) and a physical approach involving UV visualisation with a Spectroline^®^ E-Series lamp, operating at a long wavelength (254 nm), 230 V, Westbury, NY, USA. A chromatography column was assembled with silica gel (SiO_2_ Sigma-Aldrich, CAS No. 112926-00-8, St. Louis, MO, USA), using the eluents as described in Section 4.2.

Nuclear magnetic resonance (NMR) measurements were performed using a Bruker Avance DRX 300 spectrometer, with resonance frequencies of 300 MHz for ^1^H and 75 MHz for ^13^C. Deuterated chloroform (CDCl_3_ 99.8 atom % D, Sigma-Aldrich, CAS No. 865-49-6, DA, DE) was utilised as the solvent. The calibration of the spectra was accomplished by referencing the peaks to the residual solvent. High-resolution electron ionisation mass spectrometry (HREIMS) was carried out with a Bruker MAXIS II spectrometer, utilising the electrospray ionisation technique (EI^+^). Samples were directly infused at a flow rate of 3 μL min^−1^, with methanol (MeOH 99.8%, Sigma-Aldrich, CAS No. 67-56-1, St. Louis, MO, USA) containing 0.1% formic acid (HCOOH 97.5–98.5%, Sigma-Aldrich, CAS No. 64-18-6, St. Louis, MO, USA) as the ionising phase. Initial parameters included an end plate at 500 V, capillary at 3500 V, nebuliser at 0.2 bar, dry gas at 2.0 L min^−1^, dry temperature at 250 °C, and a mass range of 50–3000 Da.

### 4.2. Extraction and Isolation

Roots of *M. flexuosa* were collected in June 2019 from the Lagunas District in the Alto Amazonas Province, Loreto department, Peru (5°14′22.2″ S 75°38′30.3″ W), at an altitude of 149 m. Botanical identification was verified by the National Herbal of Medicinal Plants, National Institute of Health, CENSI, and a voucher specimen was deposited (HINS 09273).

Upon collection, the plant roots were dried in a hot air oven at 50 °C for 48 h and pulverised into a fine powder (500 g) following the protocol described by Apaza Ticona et al. [72]. This powder underwent a 30 min decoction at boiling point with 2 L of distilled water (dH_2_O). The resulting AqEMf was frozen in glass containers at −38 °C and subsequently lyophilised using a freeze dryer (Christ alpha 1e2 LD plus, Benningen, Germany) at −50 °C.

After being lyophilised, the sample (74.39 g) was extracted three times (three times/24 h/25 °C) with 500 mL of *n*-hexane (Hex, Sigma-Aldrich, CAS No. 110-54-3, St. Louis, MO, USA) at room temperature (25 ± 5 °C) for 72 h, and then evaporated in vacuo, yielding 9.71 g of the HEMf. Subsequently, three additional extractions with 500 mL (1:1 *v*/*v*) of dichloromethane (DCM, ≥99.5% Sigma-Aldrich, CAS No. 75-09-2, St. Louis, MO, USA)/MeOH were carried out under similar conditions (three times/24 h/25 °C), yielding 5.62 g of the DCMEMf. The extracts were filtered, and the solvents were removed by vacuum rotary evaporation at room temperature (25 °C). Subsequently, the extracts and fractions were stored at 4 °C, awaiting the biological assays to be carried out.

DCMEMf (5 g) was then fractionated using bio-guided SiO_2_ (40–63 μm) column chromatography (2 cm × 50 cm) with a gradual gradient of DCM/ethyl acetate (EtOAc, 99.8% Sigma-Aldrich, CAS No. 141-78-6, St. Louis, MO, USA) (6:1 → 0:1 *v*/*v*). This fractionation yielded nine fractions (F1 → F9), among which fractions F4 (184.69 mg), F6 (190.81 mg), and F7 (197.71 mg) exhibited the highest anti-inflammatory and antioxidant activities across all tested cell lines.

Subsequently, fraction F4 (180 mg) was further separated by chromatography using a SiO_2_ (40–63 μm) column (2 cm × 50 cm) with a solvent gradient of Hex/EtOAc (3:2 → 0:1 *v*/*v*). This separation produced nine sub-fractions (F4A → F4I) with in vitro anti-inflammatory and antioxidant activities. Notably, sub-fraction F4F (Compound **1**, 22.37 mg) showed the most promising results. Compound **1** was recrystallised using warm EtOAc to provide an analytical sample.

Likewise, fraction F6 (180 mg) was separated using SiO_2_ (40–63 μm) column chromatography (2 cm × 50 cm) with a solvent gradient of Hex/acetone (Ace, ≥99.8% Sigma-Aldrich, CAS No. 67-64-1, St. Louis, MO, USA) (1:1 → 0:1 *v*/*v*), resulting in seven sub-fractions (F6A → F6G) with in vitro anti-inflammatory and antioxidant activities. Among these, sub-fraction F6D (Compound **2**, 26.94 mg) had the highest activity. Subsequently, it was recrystallised using warm EtOAc to provide an analytical sample.

Finally, fraction F7 (180 mg) was isolated using SiO_2_ (40–63 μm) column chromatography (2 cm × 50 cm) with a solvent gradient of Hex/EtOAc (3:1 → 0:1 *v*/*v*), leading to two sub-fractions (F7A → F7B). Subsequently, in vitro assays were conducted to evaluate their anti-inflammatory and antioxidant activities. Notably, sub-fraction F7B (Compound **3**, 90.81 mg) had the most promising results. Subsequently, it was recrystallised using warm EtOAc to provide an analytical sample.

### 4.3. Spectroscopic Data

#### 4.3.1. 3,11-Dioxours-12-en-28-oic Acid (**1**)

White powder; [α]^25^_D_ + 96°; IR (KBr):1691, 1658 cm^−1^; ^1^H NMR (CDCl_3_): *δ*_H_ 5.64 (1H, s, H-12), 2.40 (1H, d, *J* = 11.6 Hz, H*β*-18), 1.63 (1H, s, H*α*-9), 1.31 (3H, s, H*β*-24), 1.09 (3H, s, H*α*-27), 1.02 (3H, s, H*α*-23), 1.02 (3H, s, H*β*-26), 0.98 (3H, d, *J* = 6.4 Hz, H*α*-30), 0.96 (3H, s, H*β*-25), 0.87 (3H, d, *J* = 6.8 Hz, H*β*-29), 0.87 (1H, d, *J* = 6.8 Hz, H*α*-5); ^13^C NMR (CDCl_3_): *δ*_C_ 217.2 (C-3), 199.3 (C-11), 182.3 (C-28), 163.1 (C-13), 130.7 (C-12), 60.7 (C-9), 55.3 (C-5), 52.4 (C-18), 47.7 (C-4), 47.4 (C-17), 44.5 (C-14), 43.8 (C-8), 40.0 (C-19), 38.6 (C-20), 38.5 (C-7), 38.4 (C-1), 36.7 (C-10), 36.0 (C-22), 32.3 (C-2), 30.2 (C-21), 28.4 (C-15), 26.4 (C-23), 23.6 (C-16), 21.3 (C-30), 21.0 (C-27), 20.9 (C-29), 19.0 (C-6), 18.6 (C-26), 17.0 (C-25), 15.6 (C-24); EIMS (70 eV) m/z (% rel. int.): 468 [M]^+^, HREIMS m/z [M]^+^ 468.3257 (Calculated for C_30_H_44_O_4_, 468.3240). Data were compared to the references [73].

#### 4.3.2. (3β)-3-Acetyloxy-11-oxours-12-en-28-oic Acid (**2**)

White powder; [α]^25^_D_ + 25°; IR (KBr): 3448, 1727, 1690, 1660 cm^−1^; ^1^H NMR (CDCl_3_): *δ*_H_ 5.59 (1H, s, H-12), 4.50 (1H, dd, *J* = 6.8, 4.8 Hz, H-3*α*), 2.79 (1H, dt, *J* = 6.8, 3.6 Hz, H-2*β*), 2.38 (1H, d, *J* = 11.2 Hz, H*β*-18), 2.32 (1H, m, H-9), 2.09 (1H, td, *J* = 9.2, 7.2 Hz, H*α*-2), 2.04 (3H, s, CH_3_COO^−^), 1.63 (1H, s, H*α*-9), 1.30 (3H, s, H-26), 1.29 (3H, s, H*β*-23), 1.15 (3H, s, H*α*-27), 1.14 (3H, d, 6.4 Hz, H*α*-30), 1.06 (3H, s, H*β*-26), 0.97 (3H, d, *J* = 6.4 Hz, H*β*-29), 0.93 (3H, s, H*β*-25), 0.86 (3H, s, H*α*-24), 0.86 (1H, d, *J* = 6.4 Hz, H*α*-5); ^13^C NMR (CDCl_3_): *δ*_C_ 200.0 (C-11), 182.7 (-COO^−^), 171.0 (C-28), 162.8 (C-13), 130.7 (C-12), 80.6 (C-3), 61.3 (C-9), 55.0 (C-5), 52.4 (C-18), 47.4 (C-17), 44.7 (C-14), 43.6 (C-8), 38.8 (C-19), 38.5 (C-20), 38.4 (C-4), 38.0 (C-1), 37.0 (C-10), 36.0 (C-22), 32.8 (C-7), 30.2 (C-21), 28.3 (C-15), 28.0 (C-2/C-23), 23.5 (C-16), 21.3 (C-29), 21.1 (C-27), 21.0 (CH_3_COO^−^), 19.2 (C-30), 17.2 (C-6), 17.0 (C-26), 16.7 (C-25), 16.3 (C-24); EIMS (70 eV) m/z (% rel. int.): 512 [M]^+^ (9.27). HREIMS m/z [M]^+^ 512.3507 (Calculated for C_32_H_48_O_5_, 512.7364). Data were compared to the references [74].

#### 4.3.3. (3β)-3-Hydroxy-11-oxours-12-en-28-oic Acid (**3**)

White powder; [α]^25^_D_ + 71°; IR (KBr): 3454, 1683, 1653 cm^−1^; ^1^H NMR (CD_3_OD): *δ*_H_ 5.54 (1H, s, H-12), 3.17 (1H, dd, *J* = 7.6, 4.4 Hz, H-3*α*), 2.41 (1H, d, *J* = 10.8 Hz, H*β*-18), 1.63 (1H, s, H*α*-9), 1.35 (3H, s, H*β*-23), 1.13 (3H, s, H*α*-27), 1.01 (3H, d, *J* = 6.4 Hz, H*α*-30), 0.98 (3H, s, H*β*-26), 0.89 (3H, d, *J* = 6.4 Hz, H*β*-29), 0.89 (1H, d, *J* = 6.8 Hz, H*α*-5), 0.79 (3H, s, H*β*-25); ^13^C NMR (CD_3_OD): *δ*_C_ 202.5 (C-11), 166.9 (C-13), 131.3 (C-12), 79.4 (C-3), 62.8 (C-9), 56.3 (C-5), 54.7 (C-18), 56.0 (C-17), 45.3 (C-14), 40.4 (C-4), 40.2 (C-8), 39.9 (C-19), 38.4 (C-20), 37.5 (C-1/C-22), 34.3 (C-7), 31.5 (C-21), 29.7 (C-10), 28.7 (C-15), 27.8 (C-2), 25.1 (C-16), 21.4 (C-29), 21.3 (C-27), 19.8 (C-30), 18.6 (C-6), 17.6 (C-26), 16.8 (C-25), 16.4 (C-23/C-24); EIMS (70 eV) m/z (% rel. int.): 470 [M]^+^ (7.82). HREIMS m/z [M]^+^ 470.3382 (Calculated for C_30_H_46_O_4_, 470.6987). Data were compared to the references [75].

### 4.4. Cell Culture

The IC-21 (*Mus musculus* macrophage cells, TIB-186) and RAW 264.7 (*Mus musculus* macrophage cells, TIB-71) cell lines used in this study were obtained from the American Type Culture Collection (ATCC, Manassas, VA, USA). The selection of IC-21 and RAW 264.7 cell lines for studies on inflammation and antioxidant activity is based on two main reasons: the cell type and their origin. Firstly, both IC-21 and RAW 264.7 cell lines are macrophages, which play a crucial role in the immune system, being essential for the inflammatory response and regulation of oxidative stress. These cells have the capacity to produce pro-inflammatory cytokines, express transcription factors such as NF-κB, and generate NO, making them ideal models to understand inflammatory mechanisms. Moreover, macrophages are involved in the activation of Nrf2, a transcription factor critical for the antioxidant response, allowing these cell lines to enable a comprehensive assessment of antioxidant activity within an inflammatory context [76].

Secondly, these macrophages are derived from mice, which are widely used in biomedical research due to their versatility and similarity to biological processes in humans. This origin facilitates detailed research of the molecular and cellular pathways relevant to inflammation and oxidative stress in a controlled environment, enhancing the applicability of the experimental findings to more complex models [76,77].

Cultivated in a Roswell Park Memorial Institute (RPMI) 1640 medium (RPMI 1640 Sigma-Aldrich, CAS No. R7755, St. Louis, MO, USA) supplemented with 2 mM L-glutamine (≥99% Sigma-Aldrich, CAS No. 56-85-9, St. Louis, MO, USA), 10% foetal bovine serum (FBS Sigma-Aldrich, CAS No. TMS-016, St. Louis, MO, USA), and 10,000 units of penicillin and 10 mg streptomycin mL^−1^ (Sigma-Aldrich, CAS No. P4333, St. Louis, MO, USA) in culture flasks, the cells were maintained in an incubator under normoxic conditions (20–21% O_2_) and a humidified atmosphere (5% CO_2_, at 37 °C).

Stock solutions of the samples (extracts and compounds) were prepared at a concentration of 1 mM using dimethyl sulfoxide (DMSO ≥ 99.9% Sigma-Aldrich, CAS No. 67-68-5, St. Louis, MO, USA) as the solvent. Dilutions from these stock solutions were then made to achieve concentrations between 100 and 0.20 μg mL^−1^ or μM in the culture medium, which contained 0.5% DMSO. A control group consisting only of the culture medium with 0.5% DMSO was also included to determine its potential cytotoxicity.

### 4.5. Statistical Analysis

The 50% cytotoxic concentration (CC_50_), 50% inhibitory concentration (IC_50_), and half-maximal effective concentration (EC_50_) values were calculated using the sigmoidal dose–response function. All experiments were conducted in triplicate, and the data obtained were expressed as the mean and standard deviation. A two-way analysis of variance (ANOVA) combined with Dunnett’s multiple comparisons test (*p* < 0.001) was performed for the cytotoxicity, NF-κB inhibition, NO inhibition and Nrf2 activation assays, while a one-way ordinary ANOVA combined with Dunnett’s multiple comparisons test (*p* < 0.001) was conducted for the SOD assay. All statistical analyses were performed using GraphPad Prism version 10.0.0 (La Jolla, CA, USA, www.graphpad.com, accessed on 28 August 2024).

### 4.6. In Vitro Viability Assay

Cell viability was evaluated using a colorimetric assay in 96-well plates, employing 3-amino-7-dimethylamino-2-methylphenazine hydrochloride salt reagent (NR, Sigma-Aldrich, CAS No. 553-24-2, St. Louis, MO, USA) [78]. ACTD (≥95% Sigma-Aldrich, CAS No. 50-76-0, St. Louis, MO, USA) was used as a positive control, with a CC_50_ value of 0.01 μg mL^−1^ for the extracts, or 0.008 μM for the compounds. The IC-21 and RAW 264.7 cell lines were cultured in 96-well plates at 37 °C with 5% CO_2_ until >90% confluency was achieved (1 × 10^4^ cells per well). Subsequently, the cultures were treated with sample dilutions (100–0.20 μg mL^−1^ or μM) for 72 h at 37 °C with 5% CO_2_ in phenol red-free RPMI. The supernatant was then discarded, and the cells were washed with phosphate-buffered saline (PBS) before being incubated with 100 μM NR (50 μg mL^−1^; 173 μM) for 3 h at 37 °C with 5% CO_2_. NR labelling was performed after DMSO treatment. After incubation, the labelled sample dilutions were rapidly washed with a fixative solution composed of 1% calcium chloride (CaCl_2_ Sigma-Aldrich, CAS No. 10043-52-4, St. Louis, MO, USA) and 0.5% formaldehyde solution (≥36.0% in H_2_O Sigma-Aldrich, CAS No. 50-00-0, St. Louis, MO, USA), then diluted with 100 μL of the NR extraction solution. Finally, the plates were read at 540 nm using a microplate reader (Anthos 2020, version 2.0.5, Biochrom Ltd., Cambridge, UK).

### 4.7. Anti-Inflammatory Assays

#### 4.7.1. NF-κB Inhibition Assay

To assess the anti-inflammatory potential of the samples at the same concentrations used in the cell viability assay, an NF-κB inhibition assay was used following the approach established by Apaza Ticona et al. [79]. Cells were seeded in 96-well plates at a density of 3 × 10^3^ cells per well. CEL (≥98% Sigma-Aldrich, CAS No. 34157-83-0, St. Louis, MO, USA) was utilised as a positive control, with an IC_50_ value of 3.34 μg mL^−1^ for the extracts, or 7.41 μM for the compounds. Absorbance was quantified at 450 nm using a microplate reader.

#### 4.7.2. NO Inhibition Assay

To determine the anti-inflammatory activity of the samples (at the same concentration as in the viability assay), the NF-κB inhibition assay was used, implementing the protocol adapted from Guo et al. [80]. Cells were cultured in 96-well plates (1 × 10^4^ cells per well). LNMMA (≥98% Sigma-Aldrich, CAS No. 53308-83-1, St. Louis, MO, USA) was used as the positive control, with an IC_50_ 1.61 μg mL^−1^ for the extracts, or 6.63 μM for the compounds. Absorbance was measured at 540 nm using a microplate reader.

### 4.8. Antioxidant Assays

#### 4.8.1. SOD Assay

To determine the antioxidant response of the samples (at the same concentration as in the viability assay), the SOD activation assay kit (Sigma-Aldrich, CAS No. MAK528, St. Louis, MO, USA) was used, implementing the protocol adapted from Vincent et al. [81]. GA (≥98% Sigma-Aldrich, CAS No. 149-91-7, St. Louis, MO, USA) was used as the positive control, with an EC_50_ 2.06 μg mL^−1^ for the extracts, or 12.08 μM for the compounds. Absorbance was measured at 450 nm using a microplate reader.

#### 4.8.2. Nrf2 Activity Assay

To determine the antioxidant response of the samples (at the same concentration as in the viability assay), the Nrf2 activation assay was used following the protocol carried out by Apaza Ticona et al. [79]. Cells were cultured in 96-well plates (1 × 10^4^ cells per well). CDDO-Me (≥98% Sigma-Aldrich, CAS No. 218600-53-4, St. Louis, MO, USA) was used as the positive control, with an EC_50_ 0.07 ng mL^−1^ for the extracts, or 0.11 nM for the compounds. Absorbance was measured at 540 nm using a microplate reader.

## 5. Conclusions

This study marks a significant advancement, being the first to isolate and characterise three pentacyclic triterpenoid compounds from *M. flexuosa*. This underscores the importance of investigating the therapeutic potential of this plant and its bioactive compounds.

The results evidence the promising therapeutic potential of the aqueous extract of *M. flexuosa*, particularly in terms of safety. The median cytotoxic concentrations (CC_50_) were 89.94 μg mL^−1^ for IC-21 and 98.99 μg mL^−1^ for RAW 264.7, suggesting the extract’s suitability for therapeutic use without notable cytotoxic effects.

Among the isolated compounds, compound **1** showed notable anti-inflammatory and antioxidant properties. It effectively inhibited NF-κB activation (IC_50_ between 7.39 and 8.11 μM) and NO production (IC_50_ between 4.75 and 6.59 μM) in macrophage cells. Additionally, it showed antioxidant activity by promoting SOD activation (EC_50_ 1.87 μM) and Nrf2 activation (EC_50_ between 243 and 547.59 nM), suggesting its significant therapeutic potential for inflammatory and oxidative stress-related conditions.

However, it is important to acknowledge the limitations of this study, as it relied solely on in vitro models, limiting the extrapolation of results to more complex biological systems. Future research should address these limitations through comprehensive in vivo studies.

To further explore the therapeutic potential of *M. flexuosa*, several future research directions are proposed. These include elucidating the molecular mechanisms underlying observed activities and thoroughly exploring the diversity of compounds within this plant species. Moreover, leveraging compound **1** (3,11-dioxours-12-en-28-oic acid) as a lead compound for synthesising related compounds can lead to the optimisation of its pharmacological properties, potentially helping to develop more effective and safer therapeutic agents for inflammatory and oxidative stress-related diseases through rational drug design strategies.

## Figures and Tables

**Figure 2:**
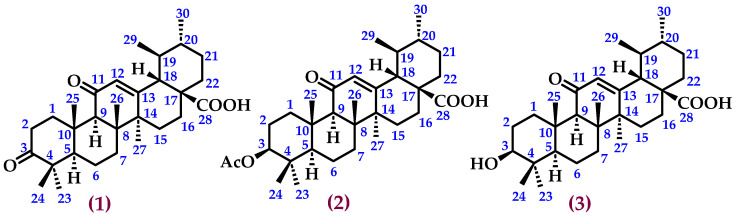
3,11-dioxours-12-en-28-oic acid (**1**), (3*β*)-3-acetyloxy-11-oxours-12-en-28-oic acid (**2**), and (3*β*)-3-hydroxy-11-oxours-12-en-28-oic acid (**3**), isolated from the DCMEMf.

## Data Availability

The datasets and materials used and/or analysed during the current study can be requested from the corresponding author upon reasonable request.

## Supplementary Materials

The following supporting information can be downloaded at: https://www.mdpi.com/article/10.3390/ijms25179615/s1.
