# Supplementary material for: Study of Pentacyclic Triterpenes from Lyophilised Aguaje: Anti-Inflammatory and Antioxidant Properties
Source: Int J Mol Sci. 2024 Sep 5;25(17):9615. doi: 10.3390/ijms25179615 (PMC11395096; doi:10.3390/ijms25179615)
Supplement: Supplementary file 1 [file ijms-25-09615-s001.zip › ijms-3190534-supplementary.pdf]

Supplementary Materials

Study of Pentacyclic Triterpenes from Lyophilised Aguaje: An-ti-inflammatory and Antioxidant Properties

Luis Apaza Ticona [1,2,*], Javier Sánchez Sánchez-Corral [2], Natalia Montoto Lozano [1], Pablo Prieto Ramos [1], and Ángel Rumbero Sánchez [2]

[1] Organic Chemistry Unit, Department of Chemistry in Pharmaceutical Sciences, Faculty of Pharmacy, University Complutense of Madrid, Plza. Ramón y Cajal s/n, 28040 Madrid, Spain
[2] Department of Organic Chemistry, Faculty of Sciences, University Autónoma of Madrid, Cantoblanco, 28049 Madrid, Spain.
* Correspondence: lnapaza@ucm.es (L.A.T). Organic Chemistry Unit, Department of Chemistry in Pharmaceutical Sciences, Faculty of Pharmacy, University Complutense of Madrid. Plza. Ramón y Cajal s/n, 28040 Madrid, Spain. ORCID: 0000-0002-7135-3909.

Contents:

- **Figure S1**. ^{1}H NMR spectrum of AqEMf in D_2O 300 MHz.
- **Figure S2**. ^{1}H NMR spectrum of HEMf in $CDCl_3$ 300 MHz.
- **Figure S3**. ^{13}C NMR spectrum of HEMf in $CDCl_3$ 75 MHz.
- **Figure S4**. ^{1}H NMR spectrum of DCMEMf in $CDCl_3$ 300 MHz.
- **Figure S5**. ^{13}C NMR spectrum of DCMEMf in $CDCl_3$ 75 MHz.
- **Figure S6**. ^{1}H NMR spectrum of 3,11-Dioxours-12-en-28-oic acid (**1**) in $CDCl_3$ 300 MHz.
- **Figure S7**. ^{13}C NMR spectrum of 3,11-Dioxours-12-en-28-oic acid (**1**) in $CDCl_3$ 75 MHz.
- **Figure S8.** HREIMS spectrum of 3,11-Dioxours-12-en-28-oic acid (**1**).
- **Figure S9**. ^{1}H NMR spectrum of (3*β*)-3-Acetyloxy-11-oxours-12-en-28-oic acid (**2**) in $CDCl_3$ 300 MHz.
- **Figure S10**. ^{13}C NMR spectrum of (3*β*)-3-Acetyloxy-11-oxours-12-en-28-oic acid (**2**) in $CDCl_3$ 75 MHz.
- **Figure S11**. HREIMS spectrum of (3*β*)-3-Acetyloxy-11-oxours-12-en-28-oic acid (**2**).
- **Figure S12**. ^{1}H NMR spectrum of (3*β*)-3-Hydroxy-11-oxours-12-en-28-oic acid (**3**) in CD_3OD 300 MHz.
- **Figure S13**. ^{13}C NMR spectrum of (3*β*)-3-Hydroxy-11-oxours-12-en-28-oic acid (**3**) in CD_3OD 75 MHz.
- **Figure S14**. HREIMS spectrum of (3*β*)-3-Hydroxy-11-oxours-12-en-28-oic acid (**3**).
- **Table S1**. Two-way analysis of variance (ANOVA) along with Dunnett's multiple comparisons test ($p < 0.001$) to assess the cytotoxicity of *M. flexuosa* extracts.
- **Table S2**. Two-way analysis of variance (ANOVA) along with Dunnett's multiple comparisons test ($p < 0.001$) to assess the cytotoxicity of *M. flexuosa* compounds.
- **Table S3**. Two-way analysis of variance (ANOVA) along with Dunnett's multiple comparisons test ($p < 0.001$) to evaluate NF-κB inhibition by *M. flexuosa* extracts.
- **Table S4**. Two-way analysis of variance (ANOVA) along with Dunnett's multiple comparisons test ($p < 0.001$) to evaluate NO inhibition by *M. flexuosa* extracts.
- **Table S5**. Two-way analysis of variance (ANOVA) along with Dunnett's multiple comparisons test ($p < 0.001$) to evaluate NF-κB inhibition by *M. flexuosa* compounds.
- **Table S6**. Two-way analysis of variance (ANOVA) along with Dunnett's multiple comparisons test ($p < 0.001$) to evaluate NO inhibition by *M. flexuosa* compounds.
- **Table S7**. Ordinary one-way analysis of variance (ANOVA) along with Dunnett's multiple comparisons test ($p < 0.001$) to evaluate the SOD assay for the *M. flexuosa* extracts.
- **Table S8**. Two-way analysis of variance (ANOVA) along with Dunnett's multiple comparisons test ($p < 0.001$) to evaluate Nrf2 activation by *M. flexuosa* extracts.
- **Table S9**. Ordinary one-way analysis of variance (ANOVA) along with Dunnett's multiple comparisons test ($p < 0.001$) to evaluate the SOD assay for the *M. flexuosa* compounds.
- **Table S10**. Two-way analysis of variance (ANOVA) along with Dunnett's multiple comparisons test ($p < 0.001$) to evaluate Nrf2 activation by *M. flexuosa* compounds.

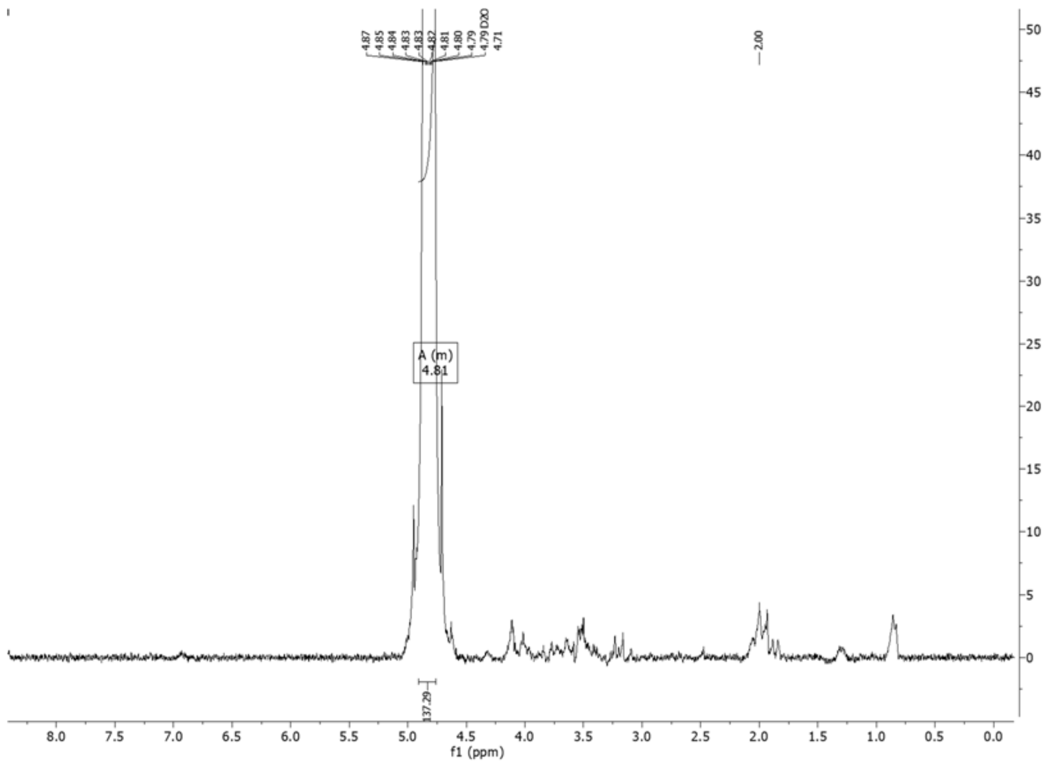

- **Figure S1**. ^{1}H NMR spectrum of AqEMf in D_2O 300 MHz.

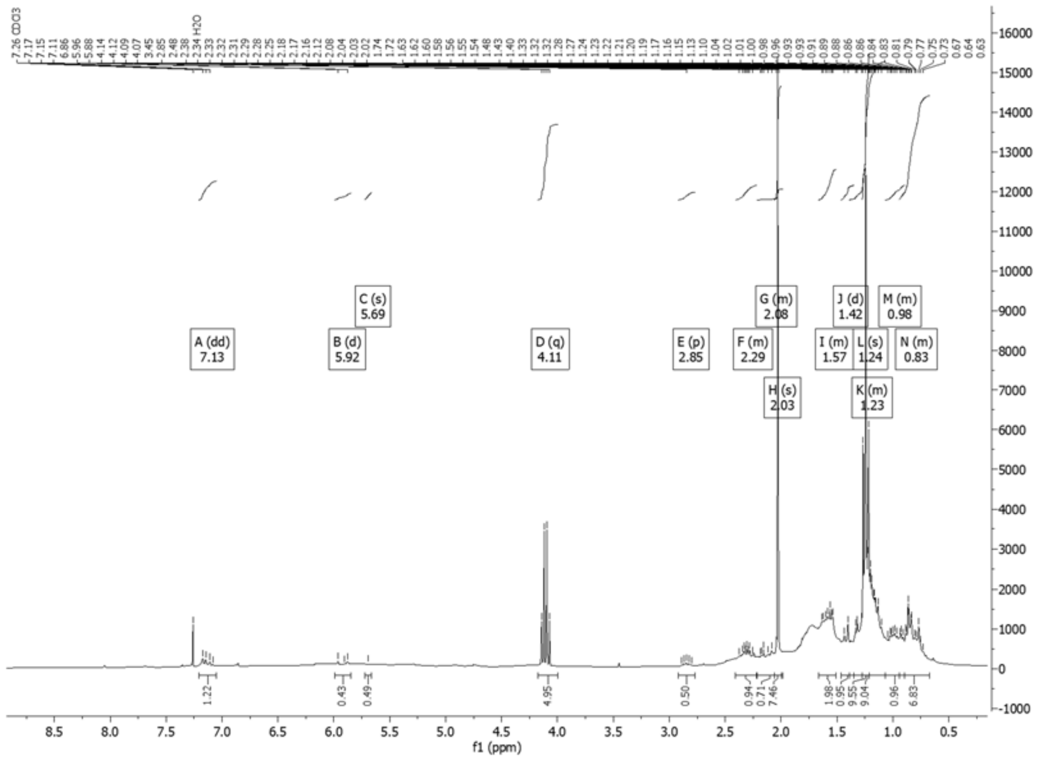

- **Figure S2**. ^{1}H NMR spectrum of HEMf in $CDCl_3$ 300 MHz.

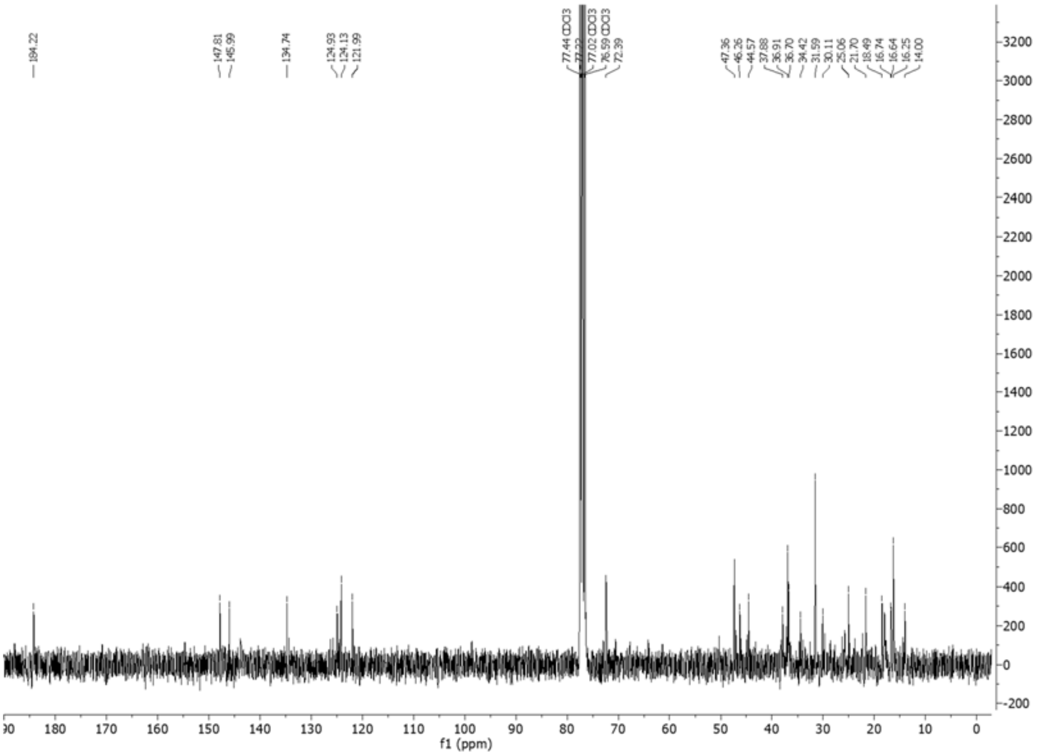

- **Figure S3**. ^{13}C NMR spectrum of HEMf in $CDCl_3$ 75 MHz.

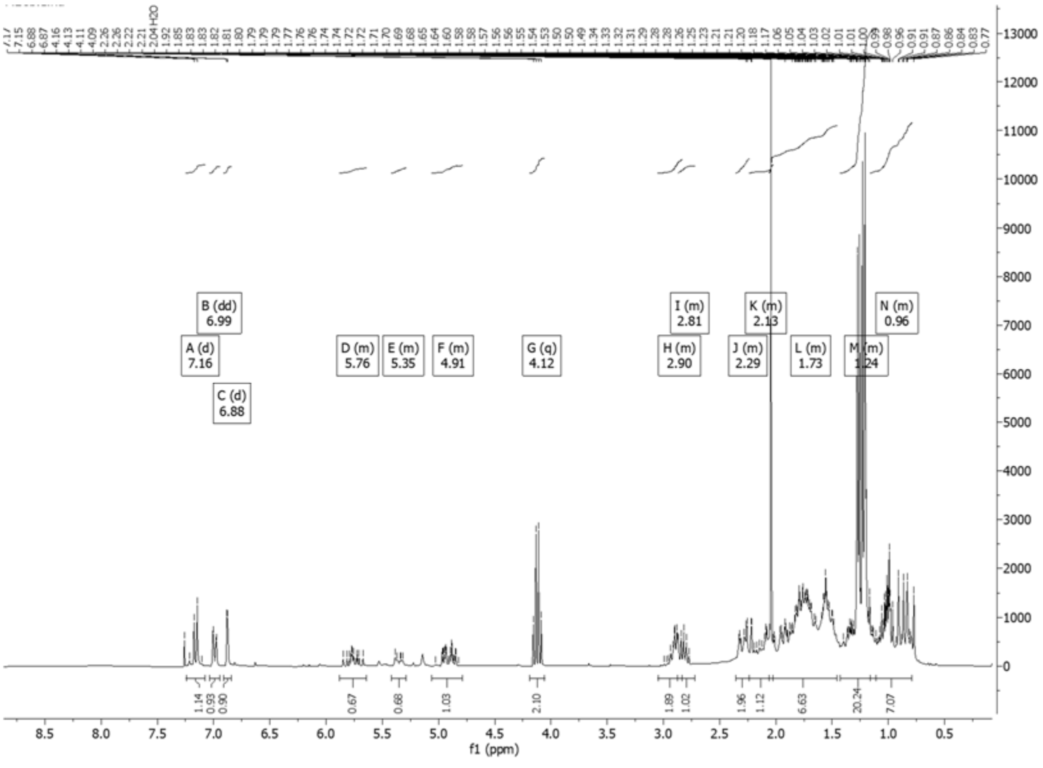

- **Figure S4**. ^{1}H NMR spectrum of DCMEMf in $CDCl_3$ 300 MHz.

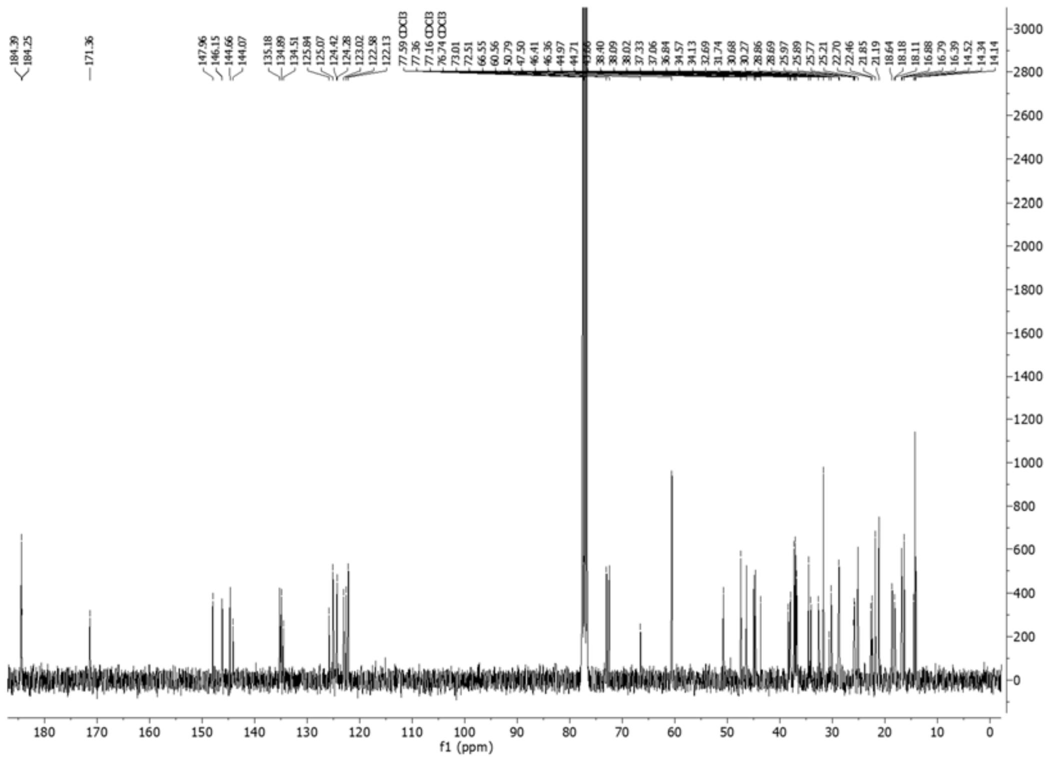

- **Figure S5**. ^{13}C NMR spectrum of DCMEMf in $CDCl_3$ 75 MHz.

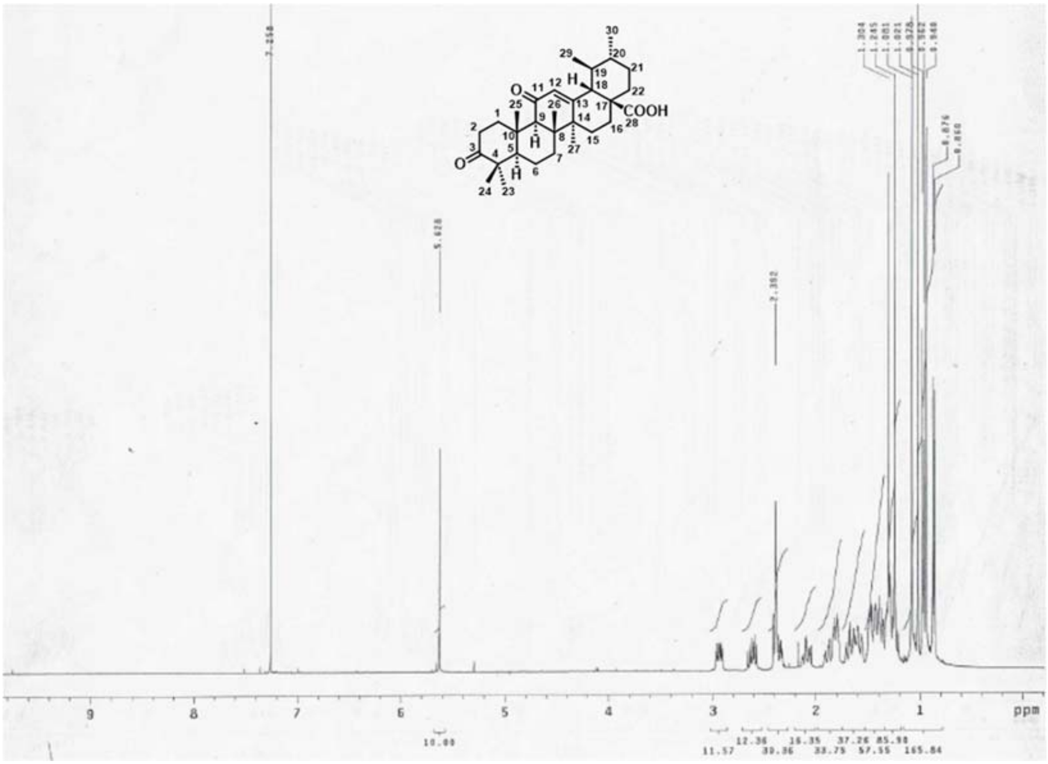

- **Figure S6**. ^{1}H NMR spectrum of 3,11-Dioxours-12-en-28-oic acid (**1**) in $CDCl_3$ 300 MHz.

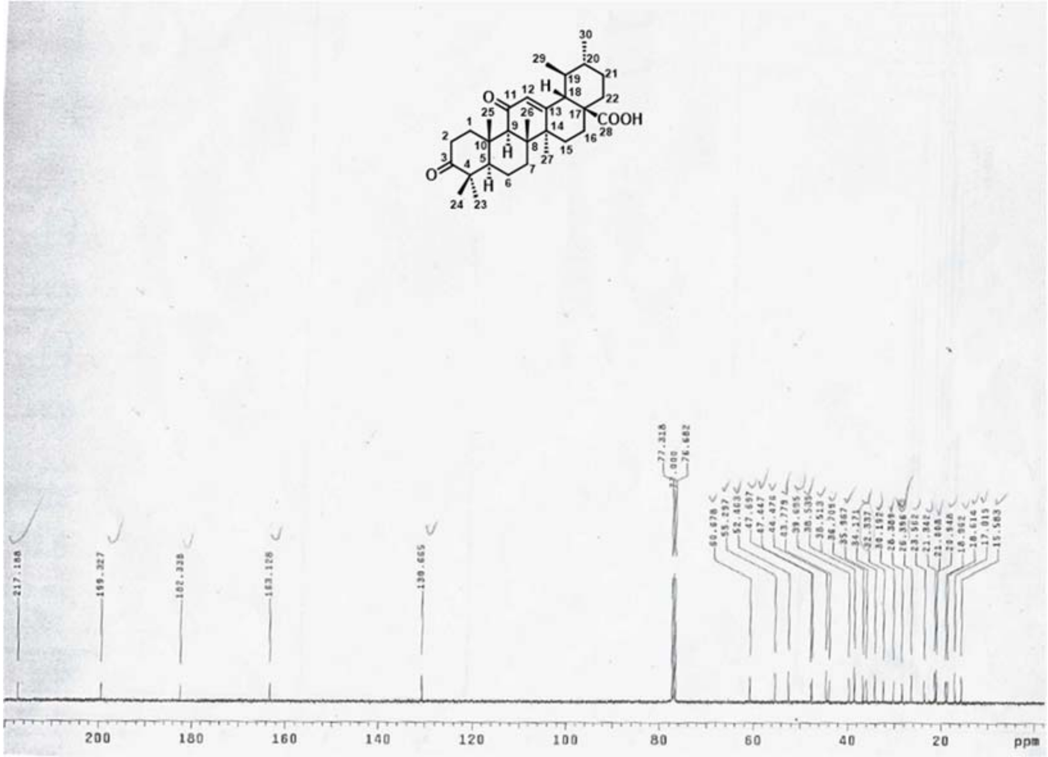

- **Figure S7**. ^{13}C NMR spectrum of 3,11-Dioxours-12-en-28-oic acid (**1**) in $CDCl_3$ 75 MHz.

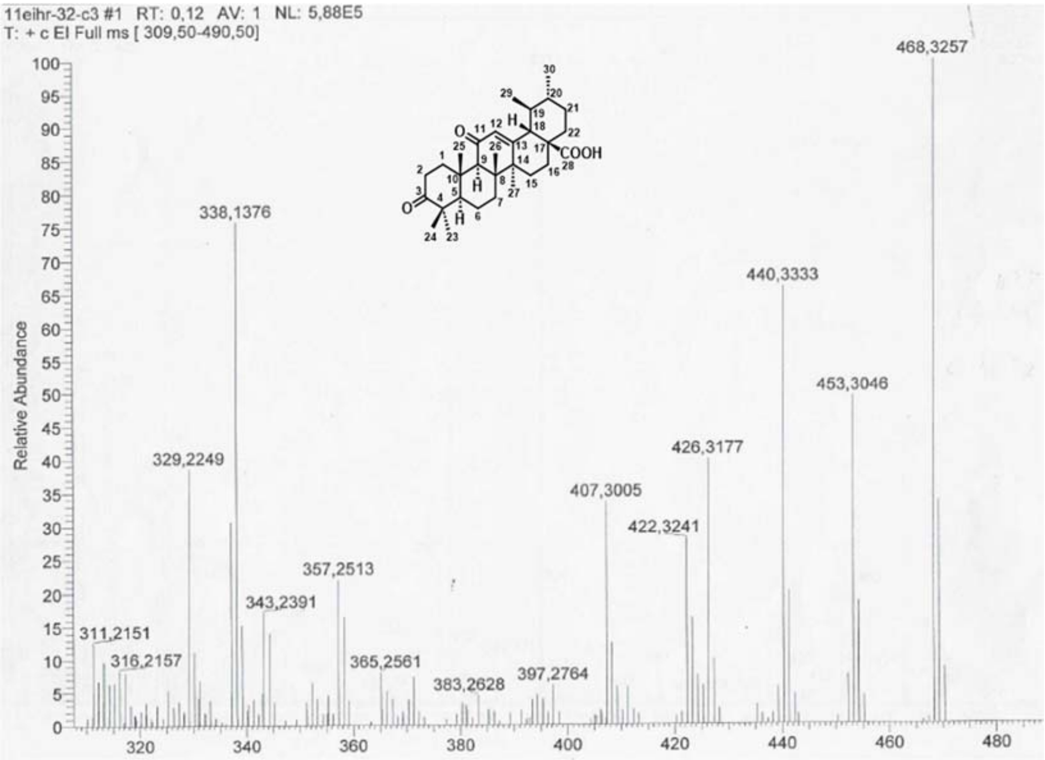

- **Figure S8**. HREIMS spectrum of 3,11-Dioxours-12-en-28-oic acid (**1**).

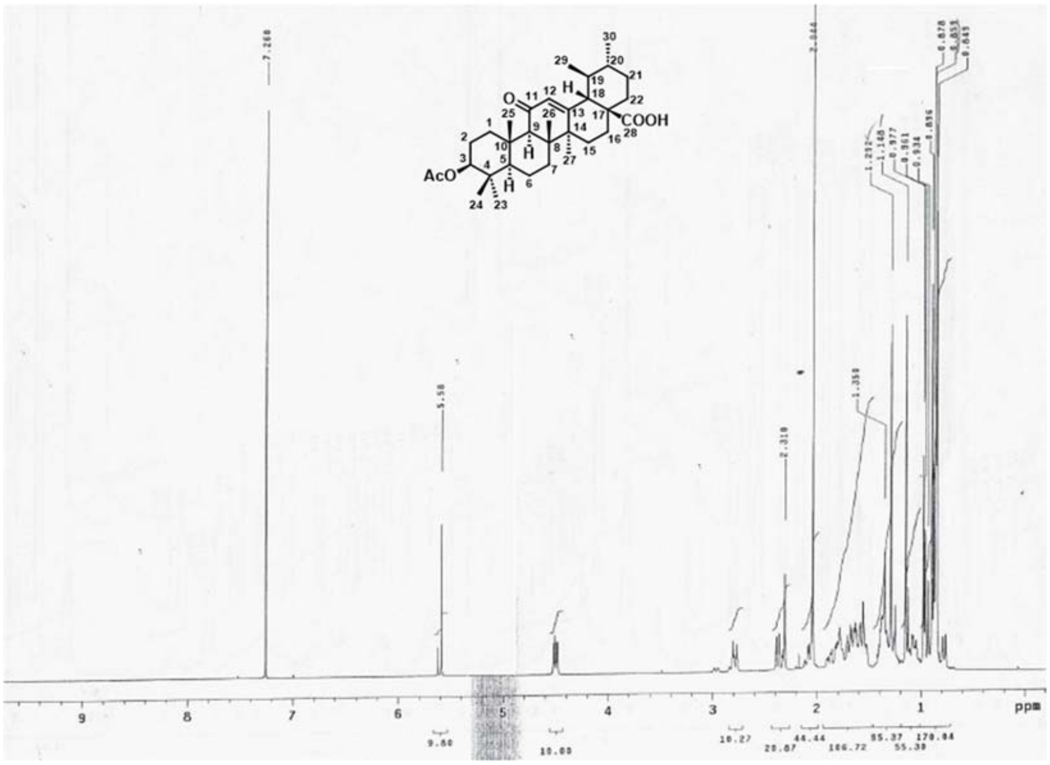

- **Figure S9**. ^{1}H NMR spectrum of (3*β*)-3-Acetyloxy-11-oxours-12-en-28-oic acid (**2**) in $CDCl_3$ 300 MHz.

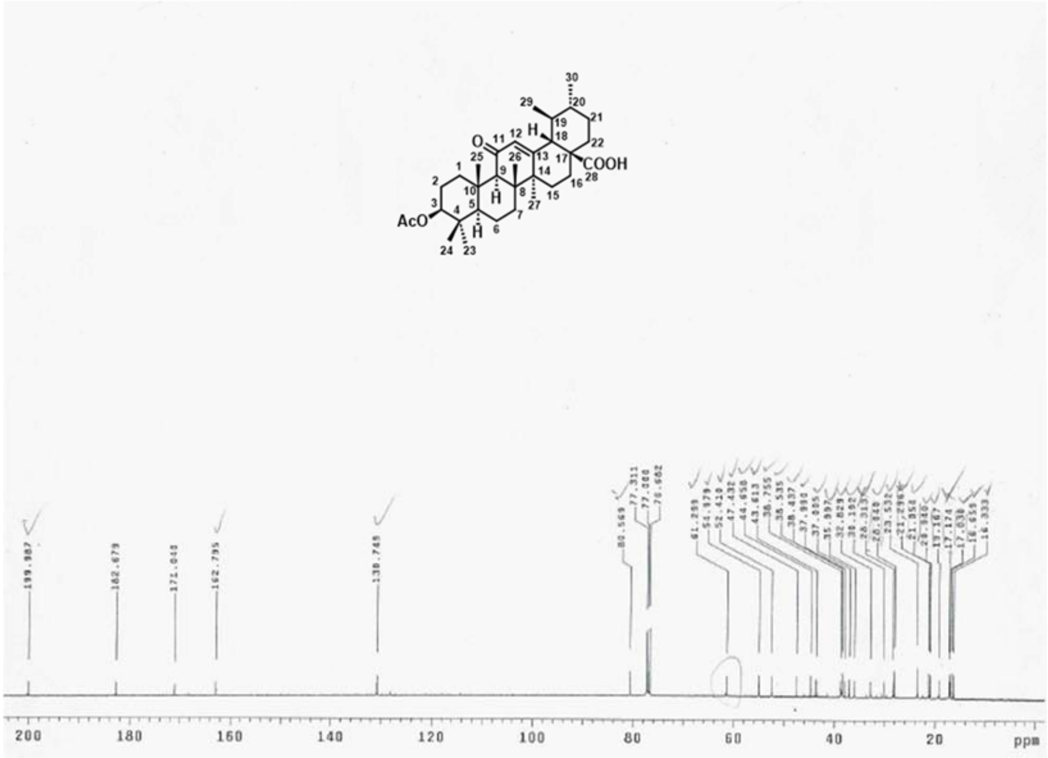

- **Figure S10**. ^{13}C NMR spectrum of (3*β*)-3-Acetyloxy-11-oxours-12-en-28-oic acid (**2**) in $CDCl_3$ 75 MHz.

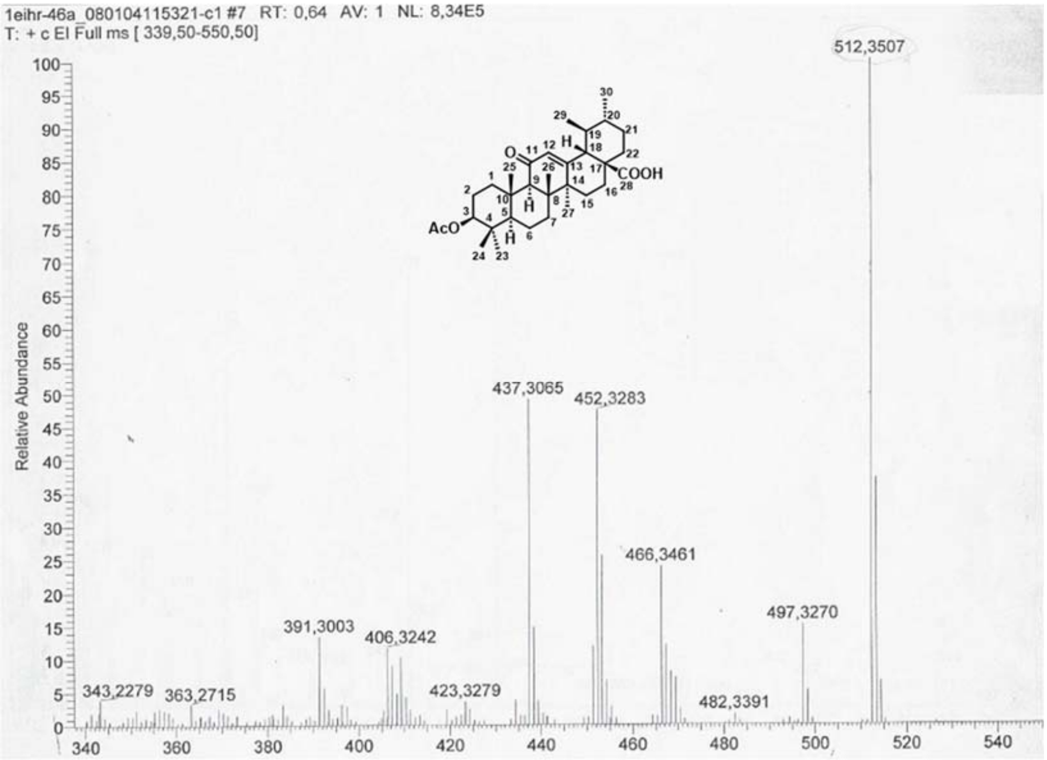

- **Figure S11**. HREIMS spectrum of (3*β*)-3-Acetyloxy-11-oxours-12-en-28-oic acid (**2**).

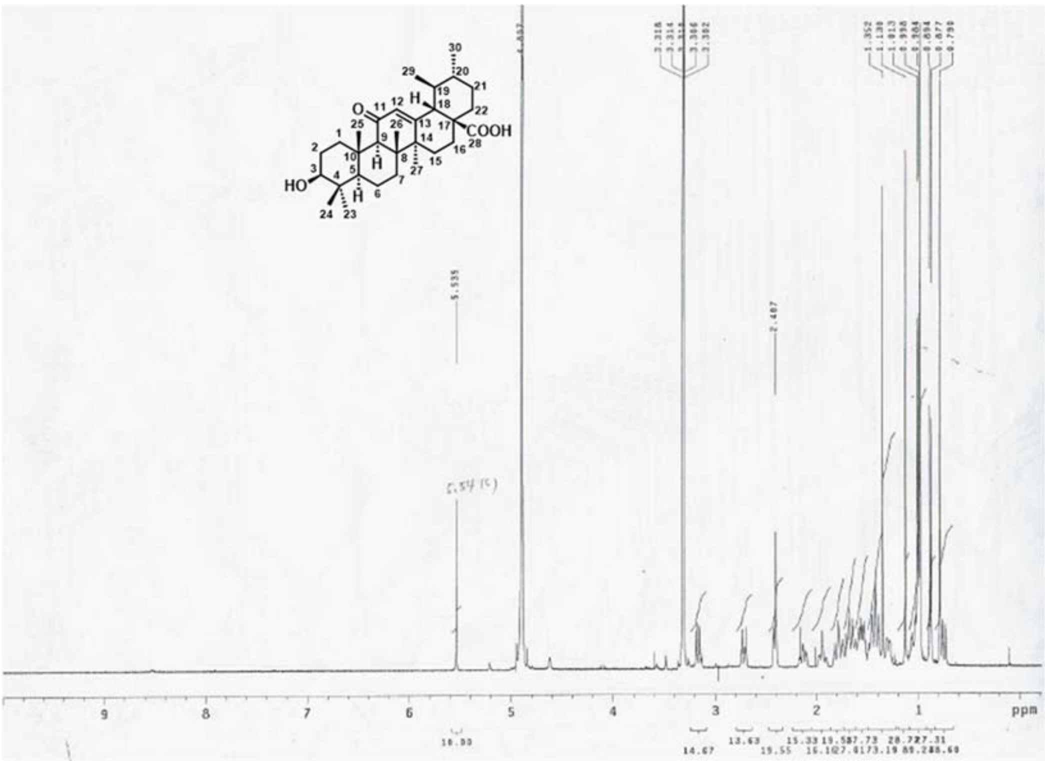

- **Figure S12**. ^{1}H NMR spectrum of (3*β*)-3-Hydroxy-11-oxours-12-en-28-oic acid (**3**) in CD_3OD 300 MHz.

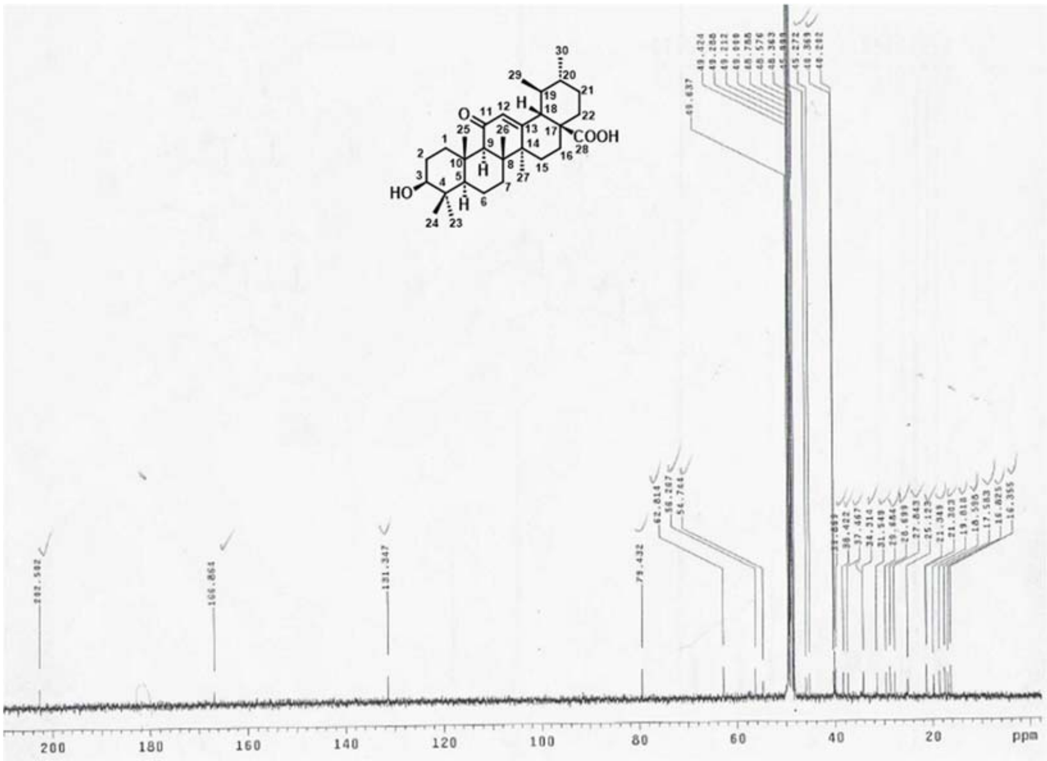

- **Figure S13**. ^{13}C NMR spectrum of (3*β*)-3-Hydroxy-11-oxours-12-en-28-oic acid (**3**) in CD_3OD 75 MHz.

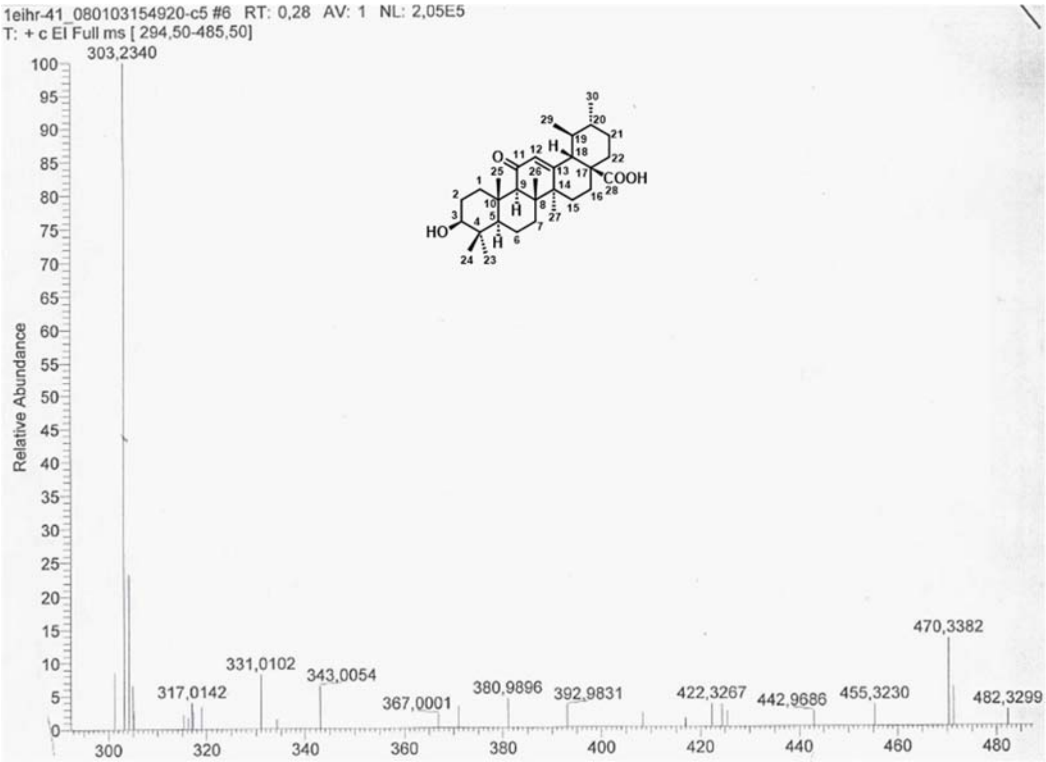

- **Figure S14**. HREIMS spectrum of (3*β*)-3-Hydroxy-11-oxours-12-en-28-oic acid (**3**).

- **Table S1**. Two-way analysis of variance (ANOVA) along with Dunnett's multiple comparisons test ($p < 0.001$) to assess the cytotoxicity of *M. flexuosa* extracts.

Table Analysed	Extracts Mf				
Two-way ANOVA	Ordinary				
Alpha	0.05				
Source of Variation	% of total variation	P value	P value summary	Significant?	
Interaction	0.497	<0.001	***	Yes	
Row Factor	0.787	<0.001	***	Yes	
Column Factor	98.6	<0.001	***	Yes	
ANOVA table	SS	DF	MS	F (DFn. DFd)	P value
Interaction	389	4	97.3	F (4. 50) = 45.1	P<0.001
Row Factor	617	1	617	F (1. 50) = 285	P<0.001
Column Factor	77250	4	19313	F (4. 50) = 8940	P<0.001
Residual	108	50	2.16		
Difference between row means					
Mean of IC-21	66.4				
Mean of RAW 264.7	72.8				
Difference between means	-6.41				
SE of difference	0.379				
95% CI of difference	-7.17 to -5.65				
Data summary					
Number of columns (Column Factor)	5				
Number of rows (Row Factor)	2				
Number of values	60				

Even though you set alpha to a value other than 0.05, one asterisk (*) identifies adjusted P values between 0.01 and 0.05, two asterisks (**) identify adjusted P values between 0.01 and 0.001, and three asterisks (***) identify adjusted P values less than 0.001, and 'ns' denotes non-significant (P > 0.05).

Within each row. compare columns (simple effects within rows)					
Number of families	2				
Number of comparisons per family	4				
Alpha	0.001				
Dunnett's multiple comparisons test	Mean Diff.	99.90% CI of diff.	Below threshold?	Summary	Adjusted P Value
IC-21					
ACTD vs. Untreated cells	-96.1	-99.4 to -92.8	Yes	***	<0.001
ACTD vs. AqEMf	-89.9	-93.3 to -86.6	Yes	***	<0.001
ACTD vs. HEMf	-64.7	-68.1 to -61.4	Yes	***	<0.001
ACTD vs. DCMEMf	-81	-84.3 to -77.6	Yes	***	<0.001

RAW 264.7					
ACTD vs. Untreated cells	-97.8	-101 to -94.4	Yes	***	<0.001
ACTD vs. AqEMf	-99	-102 to -95.6	Yes	***	<0.001
ACTD vs. HEMf	-78.8	-82.2 to -75.5	Yes	***	<0.001
ACTD vs. DCMEMf	-88.2	-91.5 to -84.9	Yes	***	<0.001

Even though you set alpha to a value other than 0.05, one asterisk (*) identifies adjusted P values between 0.01 and 0.05, two asterisks (**) identify adjusted P values between 0.01 and 0.001, and three asterisks (***) identify adjusted P values less than 0.001, and 'ns' denotes non-significant (P > 0.05).

Test details	Mean 1	Mean 2	Mean Diff.	SE of diff.	N1	N2	q	DF
IC-21								
ACTD vs. Untreated cells	0.0143	96.1	-96.1	0.849	6	6	113	50
ACTD vs. AqEMf	0.0143	89.9	-89.9	0.849	6	6	106	50
ACTD vs. HEMf	0.0143	64.8	-64.7	0.849	6	6	76.3	50
ACTD vs. DCMEMf	0.0143	81	-81	0.849	6	6	95.4	50
RAW 264.7								
ACTD vs. Untreated cells	0.0193	97.8	-97.8	0.849	6	6	115	50
ACTD vs. AqEMf	0.0193	99	-99	0.849	6	6	117	50
ACTD vs. HEMf	0.0193	78.8	-78.8	0.849	6	6	92.9	50
ACTD vs. DCMEMf	0.0193	88.2	-88.2	0.849	6	6	104	50

- **Table S2**. Two-way analysis of variance (ANOVA) along with Dunnett's multiple comparisons test ($p < 0.001$) to assess the cytotoxicity of *M. flexuosa* compounds.

Table Analysed	Compounds Mf				
Two-way ANOVA	Ordinary				
Alpha	0.05				
Source of Variation	% of total variation	P value	P value summary	Significant?	
Interaction	0.168	<0.001	***	Yes	
Row Factor	0.314	<0.001	***	Yes	
Column Factor	99.4	<0.001	***	Yes	
ANOVA table	SS	DF	MS	F (DFn. DFd)	P value
Interaction	125	4	31.3	F (4. 50) = 17.2	P<0.001
Row Factor	234	1	234	F (1. 50) = 128	P<0.001
Column Factor	73970	4	18493	F (4. 50) = 10159	P<0.001
Residual	91	50	1.82		
Difference between row means					
Mean of IC-21	66.1				
Mean of RAW 264.7	70.1				
Difference between means	-3.95				
SE of difference	0.348				
95% CI of difference	-4.65 to -3.25				

Data summary					
Number of columns (Column Factor)	5				
Number of rows (Row Factor)	2				
Number of values	60				

Even though you set alpha to a value other than 0.05, one asterisk (*) identifies adjusted P values between 0.01 and 0.05, two asterisks (**) identify adjusted P values between 0.01 and 0.001, and three asterisks (***) identify adjusted P values less than 0.001, and 'ns' denotes non-significant (P > 0.05).

Within each row. compare columns (simple effects within rows)					
Number of families	2				
Number of comparisons per family	4				
Alpha	0.001				
Dunnett's multiple comparisons test	Mean Diff.	99.90% CI of diff.	Below threshold?	Summary	Adjusted P Value
IC-21					
ACTD vs. Untreated cells	-96.1	-99.2 to -93.0	Yes	***	<0.001
ACTD vs. 1	-67.6	-70.6 to -64.5	Yes	***	<0.001
ACTD vs. 2	-78.5	-81.6 to -75.4	Yes	***	<0.001
ACTD vs. 3	-88.5	-91.6 to -85.5	Yes	***	<0.001
RAW 264.7					
ACTD vs. Untreated cells	-97.8	-101 to -94.7	Yes	***	<0.001
ACTD vs. 1	-75.4	-78.5 to -72.3	Yes	***	<0.001
ACTD vs. 2	-84.9	-88.0 to -81.8	Yes	***	<0.001
ACTD vs. 3	-92.3	-95.4 to -89.3	Yes	***	<0.001

Even though you set alpha to a value other than 0.05, one asterisk (*) identifies adjusted P values between 0.01 and 0.05, two asterisks (**) identify adjusted P values between 0.01 and 0.001, and three asterisks (***) identify adjusted P values less than 0.001, and 'ns' denotes non-significant (P > 0.05).

Test details	Mean 1	Mean 2	Mean Diff.	SE of diff.	N1	N2	q	DF
IC-21								
ACTD vs. Untreated cells	0.0085	96.1	-96.1	0.779	6	6	123	50
ACTD vs. 1	0.0085	67.6	-67.6	0.779	6	6	86.8	50
ACTD vs. 2	0.0085	78.5	-78.5	0.779	6	6	101	50
ACTD vs. 3	0.0085	88.5	-88.5	0.779	6	6	114	50
RAW 264.7								
ACTD vs. Untreated cells	0.0088	97.8	-97.8	0.779	6	6	126	50
ACTD vs. 1	0.0088	75.4	-75.4	0.779	6	6	96.8	50
ACTD vs. 2	0.0088	84.9	-84.9	0.779	6	6	109	50
ACTD vs. 3	0.0088	92.4	-92.3	0.779	6	6	119	50

- **Table S3**. Two-way analysis of variance (ANOVA) along with Dunnett's multiple comparisons test ($p < 0.001$) to evaluate NF-κB inhibition by *M. flexuosa* extracts.

Table Analysed	Extracts Mf				
Two-way ANOVA	Ordinary				
Alpha	0.05				
Source of Variation	% of total variation	P value	P value summary	Significant?	
Interaction	0.502	<0.001	***	Yes	
Row Factor	0.585	<0.001	***	Yes	
Column Factor	98.7	<0.001	***	Yes	
ANOVA table	SS	DF	MS	F (DFn. DFd)	P value
Interaction	349	5	69.7	F (5. 60) = 33.7	P<0.001
Row Factor	406	1	406	F (1. 60) = 196	P<0.001
Column Factor	68595	5	13719	F (5. 60) = 6630	P<0.001
Residual	124	60	2.07		
Difference between row means					
Mean of IC-21	48.5				
Mean of RAW 264.7	53.2				
Difference between means	-4.75				
SE of difference	0.339				
95% CI of difference	-5.43 to -4.07				
Data summary					
Number of columns (Column Factor)	6				
Number of rows (Row Factor)	2				
Number of values	72				

Even though you set alpha to a value other than 0.05, one asterisk (*) identifies adjusted P values between 0.01 and 0.05, two asterisks (**) identify adjusted P values between 0.01 and 0.001, and three asterisks (***) identify adjusted P values less than 0.001, and 'ns' denotes non-significant (P > 0.05).

Within each row. compare columns (simple effects within rows)					
Number of families	2				
Number of comparisons per family	5				
Alpha	0.001				
Dunnett's multiple comparisons test	Mean Diff.	99.90% CI of diff.	Below threshold?	Summary	Adjusted P Value
IC-21					
CEL vs. Untreated cells	-58.9	-62.2 to -55.6	Yes	***	<0.001
CEL vs. LPS	-94.3	-97.5 to -91.0	Yes	***	<0.001
CEL vs. AqEMf	-59.7	-62.9 to -56.4	Yes	***	<0.001
CEL vs. HEMf	-39.9	-43.2 to -36.6	Yes	***	<0.001
CEL vs. DCMEMf	-18.7	-22.0 to -15.4	Yes	***	<0.001

RAW 264.7					
CEL vs. Untreated cells	-61.3	-64.6 to -58.0	Yes	***	<0.001
CEL vs. LPS	-95.4	-98.7 to -92.1	Yes	***	<0.001
CEL vs. AqEMf	-72.9	-76.1 to -69.6	Yes	***	<0.001
CEL vs. HEMf	-46.5	-49.8 to -43.2	Yes	***	<0.001
CEL vs. DCMEMf	-23.8	-27.0 to -20.5	Yes	***	<0.001

Even though you set alpha to a value other than 0.05, one asterisk (*) identifies adjusted P values between 0.01 and 0.05, two asterisks (**) identify adjusted P values between 0.01 and 0.001, and three asterisks (***) identify adjusted P values less than 0.001, and 'ns' denotes non-significant (P > 0.05).

Test details	Mean 1	Mean 2	Mean Diff.	SE of diff.	N1	N2	q	DF
IC-21								
CEL vs. Untreated cells	3.24	62.2	-58.9	0.83	6	6	70.9	60
CEL vs. LPS	3.24	97.5	-94.3	0.83	6	6	114	60
CEL vs. AqEMf	3.24	62.9	-59.7	0.83	6	6	71.8	60
CEL vs. HEMf	3.24	43.2	-39.9	0.83	6	6	48.1	60
CEL vs. DCMEMf	3.24	22	-18.7	0.83	6	6	22.5	60
RAW 264.7								
CEL vs. Untreated cells	3.27	64.6	-61.3	0.83	6	6	73.8	60
CEL vs. LPS	3.27	98.7	-95.4	0.83	6	6	115	60
CEL vs. AqEMf	3.27	76.1	-72.9	0.83	6	6	87.7	60
CEL vs. HEMf	3.27	49.8	-46.5	0.83	6	6	56	60
CEL vs. DCMEMf	3.27	27	-23.8	0.83	6	6	28.6	60

- **Table S4**. Two-way analysis of variance (ANOVA) along with Dunnett's multiple comparisons test ($p < 0.001$) to evaluate NO inhibition by *M. flexuosa* extracts.

Table Analysed	Extracts Mf				
Two-way ANOVA	Ordinary				
Alpha	0.05				
Source of Variation	% of total variation	P value	P value summary	Significant?	
Interaction	0.272	<0.001	***	Yes	
Row Factor	0.435	<0.001	***	Yes	
Column Factor	99.1	<0.001	***	Yes	
ANOVA table	SS	DF	MS	F (DFn. DFd)	P value
Interaction	184	5	36.8	F (5. 60) = 17.8	P<0.001
Row Factor	294	1	294	F (1. 60) = 142	P<0.001
Column Factor	66992	5	13398	F (5. 60) = 6475	P<0.001
Residual	124	60	2.07		
Difference between row means					
Mean of IC-21	64.3				

Mean of RAW 264.7	68.3				
Difference between means	-4.04				
SE of difference	0.339				
95% CI of difference	-4.72 to -3.36				
Data summary					
Number of columns (Column Factor)	6				
Number of rows (Row Factor)	2				
Number of values	72				

Even though you set alpha to a value other than 0.05, one asterisk (*) identifies adjusted P values between 0.01 and 0.05, two asterisks (**) identify adjusted P values between 0.01 and 0.001, and three asterisks (***) identify adjusted P values less than 0.001, and 'ns' denotes non-significant (P > 0.05).

Within each row. compare columns (simple effects within rows)					
Number of families	2				
Number of comparisons per family	5				
Alpha	0.001				
Dunnett's multiple comparisons test	Mean Diff.	99.90% CI of diff.	Below threshold?	Summary	Adjusted P Value
IC-21					
LNMMA vs. Untreated cells	-64.8	-68.1 to -61.6	Yes	***	<0.001
LNMMA vs. LPS	-93.7	-96.9 to -90.4	Yes	***	<0.001
LNMMA vs. AqEMf	-82.1	-85.4 to -78.8	Yes	***	<0.001
LNMMA vs. HEMf	-71.8	-75.0 to -68.5	Yes	***	<0.001
LNMMA vs. DCMEMf	-63.8	-67.1 to -60.5	Yes	***	<0.001
RAW 264.7					
LNMMA vs. Untreated cells	-66.8	-70.0 to -63.5	Yes	***	<0.001
LNMMA vs. LPS	-95.6	-98.9 to -92.3	Yes	***	<0.001
LNMMA vs. AqEMf	-85.5	-88.8 to -82.3	Yes	***	<0.001
LNMMA vs. HEMf	-79.9	-83.2 to -76.6	Yes	***	<0.001
LNMMA vs. DCMEMf	-72.2	-75.5 to -68.9	Yes	***	<0.001

Even though you set alpha to a value other than 0.05, one asterisk (*) identifies adjusted P values between 0.01 and 0.05, two asterisks (**) identify adjusted P values between 0.01 and 0.001, and three asterisks (***) identify adjusted P values less than 0.001, and 'ns' denotes non-significant (P > 0.05).

Test details	Mean 1	Mean 2	Mean Diff.	SE of diff.	N1	N2	q	DF
IC-21								
LNMMA vs. Untreated cells	1.57	66.4	-64.8	0.83	6	6	78.1	60
LNMMA vs. LPS	1.57	95.2	-93.7	0.83	6	6	113	60
LNMMA vs. AqEMf	1.57	83.7	-82.1	0.83	6	6	98.9	60
LNMMA vs. HEMf	1.57	73.3	-71.8	0.83	6	6	86.4	60
LNMMA vs. DCMEMf	1.57	65.4	-63.8	0.83	6	6	76.8	60
RAW 264.7								

LNMMA vs. Untreated cells	1.64	68.4	-66.8	0.83	6	6	80.4	60
LNMMA vs. LPS	1.64	97.2	-95.6	0.83	6	6	115	60
LNMMA vs. AqEMf	1.64	87.2	-85.5	0.83	6	6	103	60
LNMMA vs. HEMf	1.64	81.5	-79.9	0.83	6	6	96.2	60
LNMMA vs. DCMEMf	1.64	73.8	-72.2	0.83	6	6	86.9	60

- **Table S5**. Two-way analysis of variance (ANOVA) along with Dunnett's multiple comparisons test ($p < 0.001$) to evaluate NF-κB inhibition by *M. flexuosa* compounds.

Table Analysed	Compounds Mf				
Two-way ANOVA	Ordinary				
Alpha	0.05				
Source of Variation	% of total variation	P value	P value summary	Significant?	
Interaction	0.133	<0.001	***	Yes	
Row Factor	0.247	<0.001	***	Yes	
Column Factor	99.4	<0.001	***	Yes	
ANOVA table	SS	DF	MS	F (DFn. DFd)	P value
Interaction	106	5	21.1	F (5. 60) = 9.18	P<0.001
Row Factor	197	1	197	F (1. 60) = 85.4	P<0.001
Column Factor	79257	5	15851	F (5. 60) = 6886	P<0.001
Residual	138	60	2.3		
Difference between row means					
Mean of IC-21	34.3				
Mean of RAW 264.7	37.6				
Difference between means	-3.31				
SE of difference	0.358				
95% CI of difference	-4.02 to -2.59				
Data summary					
Number of columns (Column Factor)	6				
Number of rows (Row Factor)	2				
Number of values	72				

Even though you set alpha to a value other than 0.05, one asterisk (*) identifies adjusted P values between 0.01 and 0.05, two asterisks (**) identify adjusted P values between 0.01 and 0.001, and three asterisks (***) identify adjusted P values less than 0.001, and 'ns' denotes non-significant (P > 0.05).

Within each row. compare columns (simple effects within rows)					
Number of families	2				
Number of comparisons per family	5				
Alpha	0.001				
Dunnett's multiple comparisons test	Mean Diff.	99.90% CI of diff.	Below threshold?	Summary	Adjusted P Value

IC-21					
CEL vs. Untreated cells	-53.6	-57.1 to -50.1	Yes	***	<0.001
CEL vs. LPS	-88.4	-91.9 to -84.9	Yes	***	<0.001
**CEL vs. 1**	**0.02**	**-3.44 to 3.48**	**No**	**ns**	**>0.99**
CEL vs. 2	-6	-9.46 to -2.54	Yes	***	<0.001
CEL vs. 3	-13.5	-17.0 to -10.1	Yes	***	<0.001
RAW 264.7					
CEL vs. Untreated cells	-56.2	-59.6 to -52.7	Yes	***	<0.001
CEL vs. LPS	-91.6	-95.0 to -88.1	Yes	***	<0.001
**CEL vs. 1**	**-0.48**	**-3.94 to 2.98**	**No**	**ns**	**0.97**
CEL vs. 2	-12.4	-15.8 to -8.89	Yes	***	<0.001
CEL vs. 3	-19.5	-22.9 to -16.0	Yes	***	<0.001

Even though you set alpha to a value other than 0.05, one asterisk (*) identifies adjusted P values between 0.01 and 0.05, two asterisks (**) identify adjusted P values between 0.01 and 0.001, and three asterisks (***) identify adjusted P values less than 0.001, and 'ns' denotes non-significant (P > 0.05).

Test details	Mean 1	Mean 2	Mean Diff.	SE of diff.	N1	N2	q	DF
IC-21								
CEL vs. Untreated cells	7.41	61	-53.6	0.876	6	6	61.2	60
CEL vs. LPS	7.41	95.8	-88.4	0.876	6	6	101	60
CEL vs. 1	7.41	7.39	0.02	0.876	6	6	0.0228	60
CEL vs. 2	7.41	13.4	-6	0.876	6	6	6.85	60
CEL vs. 3	7.41	21	-13.5	0.876	6	6	15.5	60
RAW 264.7								
CEL vs. Untreated cells	7.63	63.8	-56.2	0.876	6	6	64.1	60
CEL vs. LPS	7.63	99.2	-91.6	0.876	6	6	105	60
CEL vs. 1	7.63	8.11	-0.48	0.876	6	6	0.548	60
CEL vs. 2	7.63	20	-12.4	0.876	6	6	14.1	60
CEL vs. 3	7.63	27.1	-19.5	0.876	6	6	22.2	60

- **Table S6**. Two-way analysis of variance (ANOVA) along with Dunnett's multiple comparisons test ($p < 0.001$) to evaluate NO inhibition by *M. flexuosa* compounds.

Table Analysed	Compounds Mf				
Two-way ANOVA	Ordinary				
Alpha	0.05				
Source of Variation	% of total variation	P value	P value summary	Significant?	
Interaction	0.154	<0.001	***	Yes	
Row Factor	0.165	<0.001	***	Yes	
Column Factor	99.5	<0.001	***	Yes	
ANOVA table	SS	DF	MS	F (DFn. DFd)	P value
Interaction	129	5	25.9	F (5. 60) = 11.2	P<0.001

Row Factor	139	1	139	F (1. 60) = 60.4	P<0.001
Column Factor	83671	5	16734	F (5. 60) = 7269	P<0.001
Residual	138	60	2.3		
Difference between row means					
Mean of IC-21	32.9				
Mean of RAW 264.7	35.7				
Difference between means	-2.78				
SE of difference	0.358				
95% CI of difference	-3.49 to -2.06				
Data summary					
Number of columns (Column Factor)	6				
Number of rows (Row Factor)	2				
Number of values	72				

Even though you set alpha to a value other than 0.05, one asterisk (*) identifies adjusted P values between 0.01 and 0.05, two asterisks (**) identify adjusted P values between 0.01 and 0.001, and three asterisks (***) identify adjusted P values less than 0.001, and 'ns' denotes non-significant (P > 0.05).

Within each row. compare columns (simple effects within rows)					
Number of families	2				
Number of comparisons per family	5				
Alpha	0.001				
Dunnett's multiple comparisons test	Mean Diff.	99.90% CI of diff.	Below threshold?	Summary	Adjusted P Value
IC-21					
LNMMA vs. Untreated cells	-54.9	-58.3 to -51.4	Yes	***	<0.001
LNMMA vs. LPS	-90.6	-94.0 to -87.1	Yes	***	<0.001
**LNMMA vs. 1**	**1.68**	**-1.78 to 5.14**	**No**	**ns**	**0.21**
LNMMA vs. 2	-4.85	-8.31 to -1.39	Yes	***	<0.001
LNMMA vs. 3	-10.2	-13.7 to -6.75	Yes	***	<0.001
RAW 264.7					
LNMMA vs. Untreated cells	-55.3	-58.8 to -51.9	Yes	***	<0.001
LNMMA vs. LPS	-91.5	-94.9 to -88.0	Yes	***	<0.001
**LNMMA vs. 1**	**0.24**	**-3.22 to 3.70**	**No**	**ns**	**>0.99**
LNMMA vs. 2	-8.63	-12.1 to -5.17	Yes	***	<0.001
LNMMA vs. 3	-17.9	-21.4 to -14.5	Yes	***	<0.001

Even though you set alpha to a value other than 0.05, one asterisk (*) identifies adjusted P values between 0.01 and 0.05, two asterisks (**) identify adjusted P values between 0.01 and 0.001, and three asterisks (***) identify adjusted P values less than 0.001, and 'ns' denotes non-significant (P > 0.05).

Test details	Mean 1	Mean 2	Mean Diff.	SE of diff.	N1	N2	q	DF
IC-21								
LNMMA vs. Untreated cells	6.43	61.3	-54.9	0.876	6	6	62.7	60
LNMMA vs. LPS	6.43	97	-90.6	0.876	6	6	103	60
LNMMA vs. 1	6.43	4.75	1.68	0.876	6	6	1.92	60
LNMMA vs. 2	6.43	11.3	-4.85	0.876	6	6	5.54	60
LNMMA vs. 3	6.43	16.6	-10.2	0.876	6	6	11.7	60
RAW 264.7								
LNMMA vs. Untreated cells	6.83	62.1	-55.3	0.876	6	6	63.1	60
LNMMA vs. LPS	6.83	98.3	-91.5	0.876	6	6	104	60
LNMMA vs. 1	6.83	6.59	0.24	0.876	6	6	0.274	60
LNMMA vs. 2	6.83	15.5	-8.63	0.876	6	6	9.85	60
LNMMA vs. 3	6.83	24.8	-17.9	0.876	6	6	20.5	60

- **Table S7**. Ordinary one-way analysis of variance (ANOVA) along with Dunnett's multiple comparisons test ($p < 0.001$) to evaluate the SOD assay for the *M. flexuosa* extracts.

Table Analysed	SOD extracts				
Data sets analysed	A-D				
ANOVA summary					
F	3453				
P value	<0.001				
P value summary	***				
Significant diff. among means (P < 0.05)?	Yes				
R squared	0.998				
Brown-Forsythe test					
F (DFn. DFd)					
P value					
P value summary					
Are SDs significantly different (P < 0.05)?					
Bartlett's test					
Bartlett's statistic (corrected)	37.6				
P value	<0.001				
P value summary	***				
Are SDs significantly different (P < 0.05)?	Yes				
ANOVA table	SS	DF	MS	F (DFn. DFd)	P value
Treatment (between columns)	361486	3	120495	F (3. 20) = 3453	P<0.001
Residual (within columns)	698	20	34.9		
Total	362184	23			
Model comparison	SS	DF	Probability it is correct		
Null H. All population means identical	362184	23	0%		

Alternative H: Distinct population means	698	20	100%		
Ratio of probabilities			0		
Difference in AICc			141		
Data summary					
Number of treatments (columns)	4				
Number of values (total)	24				

Even though you set alpha to a value other than 0.05, one asterisk (*) identifies adjusted P values between 0.01 and 0.05, two asterisks (**) identify adjusted P values between 0.01 and 0.001, and three asterisks (***) identify adjusted P values less than 0.001, and 'ns' denotes non-significant (P > 0.05).

Number of families	1						
Number of comparisons per family	3						
Alpha	0.001						
Dunnett's multiple comparisons test	Mean Diff.	99.90% CI of diff.	Below threshold?	Summary	Adjusted P Value	A-?	
GA vs. AqEMf	-73.8	-88.4 to -59.2	Yes	***	<0.001	B	AqEMf
GA vs. HEMf	-330	-345 to -315	Yes	***	<0.001	C	HEMf
GA vs. DCMEMf	-117	-132 to -103	Yes	***	<0.001	D	DCMEMf

Even though you set alpha to a value other than 0.05, one asterisk (*) identifies adjusted P values between 0.01 and 0.05, two asterisks (**) identify adjusted P values between 0.01 and 0.001, and three asterisks (***) identify adjusted P values less than 0.001, and 'ns' denotes non-significant (P > 0.05).

Test details	Mean 1	Mean 2	Mean Diff.	SE of diff.	n1	n2	q	DF
GA vs. AqEMf	2.06	75.9	-73.8	3.41	6	6	21.6	20
GA vs. HEMf	2.06	332	-330	3.41	6	6	96.8	20
GA vs. DCMEMf	2.06	119	-117	3.41	6	6	34.4	20

- **Table S8**. Two-way analysis of variance (ANOVA) along with Dunnett's multiple comparisons test ($p < 0.001$) to evaluate Nrf2 activation by *M. flexuosa* extracts.

Table Analysed	Extracts Mf				
Two-way ANOVA	Ordinary				
Alpha	0.05				
Source of Variation	% of total variation	P value	P value summary	Significant?	
Interaction	1.39	<0.001	***	Yes	
Row Factor	1.7	<0.001	***	Yes	
Column Factor	96.9	<0.001	***	Yes	
ANOVA table	SS	DF	MS	F (DFn. DFd)	P value
Interaction	229267	4	57317	F (4. 50) = 37687	P<0.001
Row Factor	280886	1	280886	F (1. 50) = 184690	P<0.001
Column Factor	16005601	4	4001400	F (4. 50) = 2631033	P<0.001
Residual	76	50	1.52		
Difference between row means					

Mean of IC-21	426				
Mean of RAW 264.7	563				
Difference between means	-137				
SE of difference	0.318				
95% CI of difference	-137 to -136				
Data summary					
Number of columns (Column Factor)	5				
Number of rows (Row Factor)	2				
Number of values	60				

Even though you set alpha to a value other than 0.05, one asterisk (*) identifies adjusted P values between 0.01 and 0.05, two asterisks (**) identify adjusted P values between 0.01 and 0.001, and three asterisks (***) identify adjusted P values less than 0.001, and 'ns' denotes non-significant (P > 0.05).

Dunnett's multiple comparisons test	Mean Diff.	99.90% CI of diff.	Below threshold?	Summary	Adjusted P Value
IC-21					
CDDO-Me vs. Untreated cells	-1.84	-4.64 to 0.958	No	*	0.04
CDDO-Me vs. AqEMf	-1221	-1224 to -1218	Yes	***	<0.001
CDDO-Me vs. HEMf	-608	-611 to -605	Yes	***	<0.001
CDDO-Me vs. DCMEMf	-300	-303 to -297	Yes	***	<0.001
RAW 264.7					
CDDO-Me vs. Untreated cells	-1.89	-4.69 to 0.908	No	*	0.04
CDDO-Me vs. AqEMf	-1541	-1544 to -1538	Yes	***	<0.001
CDDO-Me vs. HEMf	-816	-819 to -813	Yes	***	<0.001
CDDO-Me vs. DCMEMf	-456	-459 to -453	Yes	***	<0.001

Even though you set alpha to a value other than 0.05, one asterisk (*) identifies adjusted P values between 0.01 and 0.05, two asterisks (**) identify adjusted P values between 0.01 and 0.001, and three asterisks (***) identify adjusted P values less than 0.001, and 'ns' denotes non-significant (P > 0.05).

Test details	Mean 1	Mean 2	Mean Diff.	SE of diff.	N1	N2	q	DF
IC-21								
CDDO-Me vs. Untreated cells	0.05	1.89	-1.84	0.712	6	6	2.58	50
CDDO-Me vs. AqEMf	0.05	1221	-1221	0.712	6	6	1715	50
CDDO-Me vs. HEMf	0.05	608	-608	0.712	6	6	854	50
CDDO-Me vs. DCMEMf	0.05	300	-300	0.712	6	6	421	50
RAW 264.7								
CDDO-Me vs. Untreated cells	0.08	1.97	-1.89	0.712	6	6	2.65	50
CDDO-Me vs. AqEMf	0.08	1541	-1541	0.712	6	6	2164	50
CDDO-Me vs. HEMf	0.08	816	-816	0.712	6	6	1146	50
CDDO-Me vs. DCMEMf	0.08	456	-456	0.712	6	6	640	50

- **Table S9**. Ordinary one-way analysis of variance (ANOVA) along with Dunnett's multiple comparisons test ($p < 0.001$) to evaluate the SOD assay for the *M. flexuosa* compounds.

Table Analysed	SOD compounds				
Data sets analysed	A-D				
ANOVA summary					
F	1629				
P value	<0.001				
P value summary	***				
Significant diff. among means (P < 0.05)?	Yes				
R squared	0.996				
Brown-Forsythe test					
F (DFn. DFd)					
P value					
P value summary					
Are SDs significantly different (P < 0.05)?					
Bartlett's test					
Bartlett's statistic (corrected)	1.57				
P value	0.67				
P value summary	ns				
Are SDs significantly different (P < 0.05)?	No				
ANOVA table	SS	DF	MS	F (DFn. DFd)	P value
Treatment (between columns)	481	3	160	F (3. 20) = 1629	P<0.001
Residual (within columns)	1.97	20	0.0984		
Total	483	23			
Model comparison	SS	DF	Probability it is correct		
Null H. All population means identical	483	23	0%		
Alternative H: Distinct population means	1.97	20	100%		
Ratio of probabilities			0		
Difference in AICc			123		
Data summary					
Number of treatments (columns)	4				
Number of values (total)	24				

Even though you set alpha to a value other than 0.05, one asterisk (*) identifies adjusted P values between 0.01 and 0.05, two asterisks (**) identify adjusted P values between 0.01 and 0.001, and three asterisks (***) identify adjusted P values less than 0.001, and 'ns' denotes non-significant (P > 0.05).

Number of families	1						
Number of comparisons per family	3						
Alpha	0.001						
Dunnett's multiple comparisons test	Mean Diff.	99.90% CI of diff.	Below threshold?	Summary	Adjusted P Value	A-?	

GA vs. 1	10.2	9.43 to 11.0	Yes	***	<0.001	B	1
GA vs. 2	4.65	3.87 to 5.43	Yes	***	<0.001	C	2
GA vs. 3	-1.14	-1.92 to -0.363	Yes	***	<0.001	D	3

Even though you set alpha to a value other than 0.05, one asterisk (*) identifies adjusted P values between 0.01 and 0.05, two asterisks (**) identify adjusted P values between 0.01 and 0.001, and three asterisks (***) identify adjusted P values less than 0.001, and 'ns' denotes non-significant (P > 0.05).

Test details	Mean 1	Mean 2	Mean Diff.	SE of diff.	n1	n2	q	DF
GA vs. 1	12.1	1.87	10.2	0.181	6	6	56.4	20
GA vs. 2	12.1	7.43	4.65	0.181	6	6	25.7	20
GA vs. 3	12.1	13.2	-1.14	0.181	6	6	6.3	20

- **Table S10**. Two-way analysis of variance (ANOVA) along with Dunnett's multiple comparisons test (*p* < 0.001) to evaluate Nrf2 activation by *M. flexuosa* compounds.

Table Analysed	Compounds Mf				
Two-way ANOVA	Ordinary				
Alpha	0.05				
Source of Variation	% of total variation	P value	P value summary	Significant?	
Interaction	2.22	<0.001	***	Yes	
Row Factor	2.49	<0.001	***	Yes	
Column Factor	95.3	<0.001	***	Yes	
ANOVA table	SS	DF	MS	F (DFn. DFd)	P value
Interaction	235430	4	58857	F (4. 50) = 38687	P<0.001
Row Factor	264426	1	264426	F (1. 50) = 173808	P<0.001
Column Factor	10101759	4	2525440	F (4. 50) = 1659981	P<0.001
Residual	76.1	50	1.52		
Difference between row means					
Mean of IC-21	359				
Mean of RAW 264.7	492				
Difference between means	-133				
SE of difference	0.318				
95% CI of difference	-133 to -132				
Data summary					
Number of columns (Column Factor)	5				
Number of rows (Row Factor)	2				
Number of values	60				

Even though you set alpha to a value other than 0.05, one asterisk (*) identifies adjusted P values between 0.01 and 0.05, two asterisks (**) identify adjusted P values between 0.01 and 0.001, and three asterisks (***) identify adjusted P values less than 0.001, and 'ns' denotes non-significant (P > 0.05).

Within each row. compare columns (simple effects within rows)					
Number of families	2				
Number of comparisons per family	4				
Alpha	0.001				
Dunnett's multiple comparisons test	Mean Diff.	99.90% CI of diff.	Below threshold?	Summary	Adjusted P Value
IC-21					
CDDO-Me vs. Untreated cells	-1.76	-4.56 to 1.04	No	ns	0.06
CDDO-Me vs. 1	-243	-246 to -240	Yes	***	<0.001
CDDO-Me vs. 2	-524	-527 to -521	Yes	***	<0.001
CDDO-Me vs. 3	-1026	-1029 to -1024	Yes	***	<0.001
RAW 264.7					
CDDO-Me vs. Untreated cells	-1.83	-4.63 to 0.968	No	*	0.04
CDDO-Me vs. 1	-547	-550 to -545	Yes	***	<0.001
CDDO-Me vs. 2	-772	-775 to -769	Yes	***	<0.001
CDDO-Me vs. 3	-1137	-1140 to -1135	Yes	***	<0.001

Even though you set alpha to a value other than 0.05, one asterisk (*) identifies adjusted P values between 0.01 and 0.05, two asterisks (**) identify adjusted P values between 0.01 and 0.001, and three asterisks (***) identify adjusted P values less than 0.001, and 'ns' denotes non-significant (P > 0.05).

Test details	Mean 1	Mean 2	Mean Diff.	SE of diff.	N1	N2	q	DF
IC-21								
CDDO-Me vs. Untreated cells	0.09	1.85	-1.76	0.712	6	6	2.47	50
CDDO-Me vs. 1	0.09	243	-243	0.712	6	6	341	50
CDDO-Me vs. 2	0.09	524	-524	0.712	6	6	736	50
CDDO-Me vs. 3	0.09	1026	-1026	0.712	6	6	1441	50
RAW 264.7								
CDDO-Me vs. Untreated cells	0.13	1.96	-1.83	0.712	6	6	2.57	50
CDDO-Me vs. 1	0.13	548	-547	0.712	6	6	769	50
CDDO-Me vs. 2	0.13	772	-772	0.712	6	6	1084	50
CDDO-Me vs. 3	0.13	1138	-1137	0.712	6	6	1597	50
